# Underwater image enhancement using colour balancing and morphological residual processing through gamma correction

**DOI:** 10.1038/s41598-025-33170-9

**Published:** 2026-01-14

**Authors:** Dawa Chyophel Lepcha, Bhawna Goyal, Ayush Dogra, Vivek Vullikanti, J. Albert Mayan, Prabhat Kumar Sahu, Sachin Kumar, U. Siddaraj

**Affiliations:** 1 Graduate Institute of Biomedical Informatics, College of Medical Science and Technology, Taipei Medical University, New Taipei, 235 Taiwan; 2 Department of Engineering and Technology, Marwadi University, Rajkot, Gujarat 360003 India; 3 Faculty of Engineering, Sohar University, Sohar, 311 Oman; 4 Institute of Engineering and Technology, Chitkara University, Rajpura, Punjab 140401 India; 5 Department of Computer Science & Engineering, School of Engineering and Technology, JAIN (Deemed to Be University), Bangalore, Karnataka 560069 India; 6 Department of Computer Science and Engineering, Sathyabama Institute of Science and Technology, Chennai, Tamil Nadu 600119 India; 7 Department of Computer Science and Information Technology, Siksha ‘O’ Anusandhan (Deemed to Be University), Bhubaneswar, Odisha 751030 India; 8 Department of Electronics and Communication Engineering, Galgotias College of Engineering and Technology, Greater Noida, Uttar Pradesh 201310 India; 9 Manipal Institute of Technology, Manipal Academy of Higher Education, Manipal, India

**Keywords:** Color balancing, Gamma correction, Grey-world approach, Morphological residual processing, Underwater image enhancement, Weight maps, Engineering, Mathematics and computing, Ocean sciences, Optics and photonics

## Abstract

Underwater images typically suffer from poor visibility, low contrast, and severe color distortion caused by wavelength-dependent absorption and scattering of light. These degradations not only reduce visual quality but also affect subsequent analysis and interpretation in marine and robotic imaging applications. To address these challenges, this study presents an efficient underwater image enhancement (UIE) framework that integrates color balancing, morphological residual processing, and gamma correction to achieve natural color restoration and structural enhancement. Initially, an adaptive color compensation strategy corrects the imbalance in red and blue channels, followed by morphological residual processing that refines fine textures while suppressing unwanted noise. The enhanced outputs are then fused through an adaptive multiscale fusion process guided by optimized weight maps to preserve both global illumination and local detail. A final gamma correction step ensures perceptually balanced contrast and brightness. The proposed method requires no training data or prior depth estimation making it computationally efficient and robust for real-time applications. Extensive experiments conducted on multiple benchmark underwater datasets demonstrate that the proposed approach consistently outperforms 22 state-of-the-art UIE techniques in both qualitative and quantitative assessments. The method achieves superior results in terms of peak signal-to-noise ratio (PSNR), structural similarity index measure (SSIM), underwater image quality measure (UIQM), and underwater color image quality evaluation (UCIQE) metrics, confirming its capability to restore realistic colors, enhance visibility, and preserve fine details. The proposed framework provides an effective and lightweight solution for practical underwater imaging enhancement. This work supports SDG 14 (Life Below Water) by enhancing underwater imagery for marine monitoring, SDG 9 (Industry, Innovation and Infrastructure) through an efficient real-time imaging framework, and SDG 12 (Responsible Consumption and Production) by enabling accurate underwater inspection that promotes sustainable resource use.

## Introduction

Underwater imaging has been a significant and extensively explored domain for several decades, driven by the abundant resources found in aquatic environments such as rivers, lakes, and oceans^1^. Underwater image processing presents greater challenges than image processing in terrestrial environments due to the difficult physical characteristics of the aquatic environment. Moreover, color distortion and contrast reduction often occur in underwater photographs due to light scattering and absorption in the water^2^. In an underwater optical imaging model, the acquired light comprises three principal components: (a) forward scattering component, which is characteristic of light reflected from the objects and dispersed at different angles; (b) backward scattering component derived from light reflected by suspended particles rather than the target object; and (c) direct component, which pertains to light reflected from the object without experiencing scattering in water. The three elements can be perceived as a linear superposition in an underwater image. Backward scattering component conceals the edges and details of underwater images, whereas the forward scattering component results in fuzzy structures. The color distortion in underwater images is primarily due to the differential absorption of different wavelengths^3–6^. Red light, being the longest wavelength and lowest energy, attenuates at different rates compared to other light wavelengths. This indicates that green and blue light reduce at varying rates compared to red light. This attribute imparts a bluish or greenish color to underwater photographs. The scattering phenomenon randomly alters the trajectory of light propagation, whereas absorption significantly reduces light energy^3–6^. The underwater characteristics ultimately lead to color shift and contrast deterioration in underwater photographs, which is crucial for generating high quality underwater image for subsequent processing^7^.

This study presents a robust method aims at enhancing the visual quality of underwater images using color balance and morphologically processed residuals. The proposed method initiates with a low-pass linear filter integrated with morphologically processed residuals to enable edge aware processing of the input photographs. The proposed method selectively enhances important image areas while maintaining essential edge features through the integration of linear low pass filtering and nonlinear approaches. Furthermore, our approach employs numerous inputs to produce two principal images: one through contrast adjustment and the other via enhancement of a color-balanced rendition of the original image. The color balancing process successfully alleviates the color induced by underwater light scattering yielding a more natural and aesthetically pleasing image. We utilize a multi-scale fusion technique to guarantee seamless integration of upgraded characteristics while avoiding the introduction of artifacts. An essential benefit of our method is its capacity to reliably generate superior results without necessitating parameter optimization, attributable to the meticulously crafted improvement framework. The primary contributions of the proposed work are as follows:

1. This study presents a highly robust and efficient approach for underwater image enhancement (UIE). Detailed experiments highlight the remarkable effectiveness of our method, which not only achieves rapid processing speeds but also proves beneficial for various underwater vision applications. Besides, our method demonstrates strong generalization capabilities by effectively enhancing images captured in hazy and sandstorm conditions.
2. We use nonlinear filtering to detect and extract prominent edges for integration into the final image while ensuring artifact-free regions while significantly improving the visual quality of the fused image.
3. We introduce an adaptive colour correction method that minimizes colour loss and employs a fusion strategy guided by maximum attenuation mapping. Unlike conventional colour correction methods, our approach preserves colour integrity by accounting for the attenuation properties across multiple colour channels while resulting in more natural and visually accurate corrected images.
4. We introduce an adaptive contrast enhancement strategy that utilizes internal and squared integral maps to precisely estimate the mean and variance of local image regions while enabling precise and effective contrast adjustment. In addition, an efficient colour correction is used to mitigate colour distortion in underwater images by ensuring superior colour restoration and enhanced visual naturalness compared to traditional colour constancy methods.

Unlike most contemporary underwater enhancement techniques that rely heavily on deep neural networks trained on large-scale paired or synthetic datasets, the proposed method provides a non-learning yet high-performing alternative that emphasizes computational transparency, interpretability, and low-cost deployment. The algorithm’s design leverages physically interpretable modules such as color balancing based on wavelength attenuation, morphological residual processing for structure refinement, and multiscale fusion for perceptual integration without requiring any data-driven parameter tuning or pretraining. This makes the framework particularly suitable for real-time underwater imaging systems, resource-constrained hardware, and environments lacking sufficient labeled data. Moreover, the proposed approach achieves competitive visual quality with deep models while maintaining a significantly lower computational footprint, thereby contributing a practical, explainable, and efficient alternative to deep learning–dominated paradigms.

## Related works

In recent years, numerous techniques for underwater restoration and enhancement have been developed for enhancing the visibility and clarity of degraded underwater photographs. The detailed analyses of recent existing methodologies are presented below in three different groups.

### Restoration-based method

An initial category is the image restoration techniques that enhancement underwater photographs through the application of restoration models^8,9^. Given that underwater photographs often appear foggy, numerous dehazing techniques have been utilized to improve compromised underwater images^10^. Han et al.^11^ established an extensive underwater image repository known as the Heron islandcoral reef dataset (HICRD) to assess current methodologies and foster the advancement of new deep-learning algorithms. They utilized a particular water property (diffuse attenuation coefficient) to produce the reference photographs. The unpaired training dataset comprises 2000 reference repaired photographs and 6003 original underwater photographs. Furthermore, the method introduces an approach for underwater restoration through an unsupervised image to image translation network. Zhang et al.^12^ present an underwater color image processing method that integrates spatial and frequency domains, which enhances image contrast in the frequency domain by adaptively adjusting image color in the spatial domain, and finally combining the contrast enhanced image with a color-corrected version in the CIEL*a*b* color space. Sharma et al.^13^ demonstrated a method indicating that choosing the ideal receptive field dimension according to traversal range of the color channel can significantly enhance performance in the job of underwater image restoration (UIR). Moreover, it is essential to minimize extraneous multi-contextual variables and enhance the model’s representational capacity. Consequently, this system integrated a precise skip system for adaptably enhance a learned multi-contextual information. The architecture termed as Deep WaveNet is optimised using conventional pixel-to-pixel cost function.

Zhou et al.^14^ present a dehazing technique that employs an enhanced model utilizing the scene depth map and a color correction scheme to mitigate color distortion. It initially establishes a method for determining the depth of underwater photographs to generate the depth map. Thereafter, backscatter is evaluated and eliminated by the channel related to updated algorithm depends on the depth value of each pixel. The system incorporates an automated color correcting process to modify the overall color distribution of the image. The authors introduce an underwater image restoration approach referred to as the multi-scale contrast and luminance adjustment (MCLA) method^15^. The approach presents an adaptive BLEU through multicolour model conversion, efficiently removing color casts while reducing interferences from white objects and suspended particles. This research presents an advanced depth-map estimation technique that employs feature priors to enhance detail and restore features. Subsequently, it modifies the red channel using a shorter wavelength to rectify the depth map and generate a transmission map in accordance with the Lambert–Beer rule. Luo et al.^16^ proposed a fusion-based method aimed at restoring and enhancing underwater images. Their approach integrates color balancing, contrast enhancement, and histogram stretching to improve visual quality. To address the common issue of color shift in underwater scenes, the method adjusts the scalar values of the red, green, and blue channels such that their histogram distributions become more aligned, thereby achieving a more natural and balanced color representation. An advanced contrast algorithm is utilized to ascertain the optimal transmittance rather than modifying the transmittance in dark channel prior-based restoration. To improve the brightness as well as contrast of underwater photographs, a histogram stretching technique based on the red channel is employed. Wang et al.^17^ introduced modeling underwater color disparities that helps to achieve more natural color consistency. Their disparity-aware correction improves perceptual balance across varying conditions.

Zhang et al.^18^ proposed an UIE method that leverages transfer learning and consists of two key components: a domain transformation module and an image enhancement module. These modules collaboratively perform color correction and visual enhancement, while also adapting in-air image dehazing algorithms for underwater scenarios. For maintaining the physical realism of underwater scenes, the framework incorporates a physical image formation method into the domain transformation module, ensuring that the enhanced outputs remain consistent with the inherent properties of underwater imagery. The coarse-grained similarity computation is incorporated to the domain transform module to effectively eliminate color variation and enhance model performance. Hou et al.^19^ proposed a variational framework for the restoration of underwater images affected by non-uniform illumination, utilizing the illumination channel sparsity prior (ICSP). The illumination channel sparsity prior is based on the fact that the illumination channel of a uniformly illuminated underwater images in hue, saturation, and intensity (HIS) color space comprises a limited number of pixels with low intensity. Subsequently, they formulate a variational model based on a Retinex theory, incorporating a L0 norm, a constrained terms and gradient term by combining the proposed intensity and chromaticity separation processing (ICSP) into an enhanced underwater image generation model. These three regularisations noticeably enhance brightness, rectify color distortion, show structures and complex details. Concurrently, it utilizes an efficient numerical approach grounded in the alternating direction method of multipliers (ADMM) to expedite the resolution of this optimisation problem. Song et al.^20^ introduce a UIE model and proposes a dynamic technique by employing a 3D lookup table. This technology surpasses the limitations of conventional techniques dependent on the classical underwater enhancement methods and adeptly addresses the difficulties presented by the complex water and lighting conditions.

### Enhancement-based method

The second group comprises image enhancement algorithms^21–23^ that produce visually pleasing underwater photographs by modifying pixel values without the necessity of physical models^24–29^. Zhang et al.^30^ proposed a squared integral image mapping for efficiently estimating the mean and the variance of local image regions, which are then utilised for adaptively adjust the contrast of the original image. In addition, a color balancing technique is applied for correcting color inconsistencies between *a* and *b* channels in the CIELAB color space, resulting in a more visually balanced image. A robust technique is introduced utilizing multi-interval sub-histogram perspective equalization to tackle the challenges associated with underwater photography^31^. It assesses the extent of feature drifts in various regions of an image by examining its statistical properties, utilizing this data to inform feature enhancement for adaptive improvement, hence enhancing the visual quality of degraded photographs. Fu et al.^32^ present a probabilistic network to determine the enhancement of degraded underwater photographs. It integrates a conditional variational autoencoder with adaptive instance normalisation to define the enhancement distribution. It subsequently employs a consensus method to forecast a deterministic result based on a collection of samples from the distribution. This strategy can partially mitigate the bias proposed in the labelling of the reference map by analysing the enhancement distribution. The consensus method yields a robust and consistent outcome. Dual histogram iterative thresholding and a constrained histogram method utilizing Rayleigh distribution are used to boost both global and local contrast of the color corrected image, resulting in a globally improved version and a locally enhanced version, respectively^33^. A multiscale fusion technique is used to merge the complementing advantages of the globally contrast enhanced version and the locally contrast-enhanced version. They finally device multiscale unsharp masking technique to improve the fused image for enhancing visual quality.

Zhou et al.^34^ devised a multi-frame UIE technique through a multi-frame enhancement framework (MFEF). The quality of reconstruction results depends on the input image quality and requires pre-processing to achieve high quality images hence enhancing the final reconstruction output. The text discusses the color balancing technique and the contrast-limited adaptive histogram equalization (CLAHE) method, which utilize multiple input pathways to extract a wide range of intricate features from diverse perspectives. They devised a technique utilizing light scattering properties^35^. The color cast is categorised into several groups based on the average ratios of the RGB channels. The authors employ optimal attenuation characteristics to assess the color degradation rate of the RGB channels across multiple underwater scenes, and proposed a multi-scene color-restoration technique to correct the resulting color distortions. The 64 block multi contrast factor histogram stretching technique was used for enhancing the contrast of the underwater image while maintaining a uniform color. Lin et al.^36^ proposed a method to enhance underwater images that integrates adaptive color correction with an enhanced Retinex method. This technique is a single-image enhancing procedure that requires neither specialist equipment nor prior understanding of underwater settings. Initially, adaptive color correction is used to solve the color cast issue in distorted underwater photographs. The method employs image decomposition to enhance the detail component and yield a detail enhanced image. Chen et al.^37^ introduced a domain adaptation system for UIE that separates information and style representations. This method aims for separating encoded features into content and style latent variables, enabling effective style differentiation across domains. The enhancement and domain adaptation are performed within the latent space, and a user interface is provided to allow interactive adjustment of enhancement levels through latent manipulation. A novel framework is proposed in^38^ that integrates variational methods with pyramid-based processing to enhance image quality in the frequency domain. The framework introduces two variational models: an adaptive variational contrast enhancement (AVCE) model and the complete Laplacian model. These models are designed to enhance foreground contrast while preserving multi-scale textural details. To efficiently solve these models, the framework employs two optimization strategies; a gradient descent method (GDM) model and the alternating direction method of multipliers (ADMM). Furthermore, the Fast Fourier transform (FFT) is utilized to accelerate computation and enhance processing efficiency.

Zhuang et al.^39^ proposed a retinex variational model with hyper Laplacian reflectance priors for enhancing underwater imagery. Hyper-Laplacian reflectance priors are defined by the ${{\ell}}_{1/2}$-norm penalty imposed on the first and second order gradients of the reflectance. This employs sparsity promoting methods and extensive reflectivity to enhance significant structures and intricate details while maintaining the veracity of natural colors. Furthermore, the ${{\ell}}_{1/2}$-norm is deemed appropriate for precisely measuring light. Subsequently, they split a complex UIE problem into manageable subproblems that concurrently and independently assess reflection and lighting, which are utilized to improve underwater photographs within a retinex variational model. Wang et al.^40^ introduced a reinforcement learning framework that autonomously determines several image enhancement techniques and optimizes their parameters for the improvement of underwater photographs. Unlike end-to-end deep learning black box systems, the model functions as a white-box, providing transparency in method selection and parameter setup operations. This technique integrates human visual perception and existing knowledge of underwater color with non-reference score adjustments to increase underwater image quality. This exceeds the training limitations established by volunteer-selected enhanced photographs as standards. A hybrid fusion method (HFM) for enhancing underwater images is proposed in^41^, which incorporates a color balance correction module that addresses color distortions by leveraging the gray-world assumption alongside a nonlinear color mapping function. Additionally, it introduces a visibility recovery module based on type-II fuzzy sets, and a contrast enhancement module that utilizes curve transformation techniques to improve the overall visual quality of underwater photographs. Also, the authors introduced an underwater image perceptual fusion model that simultaneously addresses two distinct objectives: enhancing the visibility and the contrast of underwater images. The proposed technique adeptly addresses issues of color balance distortion, color shift, limited visibility, and inadequate contrast in underwater images, while demonstrating enhanced performance in geometric rotation estimation, feature point matching and edge recognition assessments. Wang et al.^42^ proposed a UIE architecture comprises of three phases: meta submergence (metamergence), meta relief (metalief), and meta ebb (metaebb). These phases focus on virtual underwater image synthesis, underwater image depth map estimation, and the development of advanced physical models for UIE via reinforcement learning. The initial phase supplies essential data for the training of subsequent phases, which are conducted separately. The methodology designates the three stages as metalantis due to its training methodologies, which encompass transitions from submersion to elevation and flow inside indoor environments, while emulating the virtual expanses of Atlantis. Goyal et al.^43^ proposed an image improvement technique utilizing a rigorous dictionary learning approach and a color restoration module (CRM). It integrates dictionary learning with edge aware filter based on the detail enhancement. It ensures that each minor detail patch can be sufficiently represented in the complete details derived from several training detail patches by iterative ${{\ell}}_{1}$-norm minimisation. Dictionary learning has effectively addressed several challenges in detail enhancement by eliminating the inherent constraint of training detail patches within the refined detail patches.

### Deep learning-based method

Deep learning has achieved significant success in low-level visions tasks, including image dehazing^44,45^, super-resolution^46–48^, and fusion^49,50^, owing to its robust computing capabilities and ample training data^51^. Simultaneously, significant advancements in engineering have been achieved using many deep learning based neural network methodologies^52,53^. Deep learning methodologies can enhance underwater image quality in various scenarios^54,55^. Peng et al.^56^ developed a large-scale underwater image (LSUI) dataset that encompasses a wider variety of underwater habitats and superior quality reference photographs compared to recent underwater datasets. Dataset comprises 4279 real world underwater image pairs each containing associated pairs of raw images, clear reference images, semantic segmentation maps, and medium transmission maps. The authors also proposed a color multi-scale fast Fourier transform (CMSFFT) module and a structure-guided frequency mapping and transformation (SGFMT) module specifically constructed for the UIE task, thereby enhancing the network’s focus on color channels and spatial regions with significant attenuation. Sun et al.^57^ proposed a reinforcement learning-based framework for UIE by formulating the task as a Markov decision process (MDP). In this formulation, image feature maps represent states, enhancement techniques serve as actions, and rewards correspond to improvements in image quality. A deep Q-network guides the selection of enhancement actions at each step, transitioning the image from one state to another. The process continues until the image achieves the highest cumulative quality improvement, resulting in the final enhanced output. Building on domain adaptation strategies, Wang et al.^58^ introduced a two-phase underwater domain adaptation network (TUDA) to reduce both inter-domain and intra-domain discrepancies. In the first phase, a triple-alignment network is used, which consists of a translation module to increase image realism and a task-specific enhancement module. These components apply adversarial learning at the image, feature, and output levels to promote domain invariance. In the second phase, real-world underwater images are categorized into simple and complex groups based on enhancement quality, using a rank-based underwater quality assessment algorithm. Lyu et al.^59^ introduced a simple yet efficient convolutional neural network (CNN)-based approach for improving underwater images. This methodology comprises two phases: a CNN based enhancement and YUV-based post-processing. A lightweight CNN architecture is constructed for extracting latency information from the input image for enhancement. Jiang et al.^60^ introduced an UIE method that obviates the necessity for training on synthetic underwater photographs and is independent of underwater ground truth photographs. The domain adaptation model is introduced for enhancing real world underwater photographs using transfer learning, employing in-air image dehazing techniques for UIE.

Liu et al.^61^ presented an adaptive learning attention network termed as LANet, aimed at improving underwater photographs using supervised learning to address degradation challenges. The multiscale fusion module is employed to integrate several spatial information. Secondly, they develop a typical pixel attention module (PAM) to emphasize lighted elements and enhance color information in combination with pixel and channel attention. An attention learning module (ALM) can retain inconsequential information while selectively acquiring critical feature information. This approach employs a multinomial loss function that combines mean absolute error with perceptual loss. They implement an asynchronous training model for enhancing the network’s performance concerning the multinomials loss function. Sun et al.^62^ presented an underwater multiscale generative adversarial network (GAN) designed to improve underwater photography. The network enables unpaired image to image translation between turbid and clear underwater environments. It significantly enhances multiple genres of underwater photography. The feedback systems and a noise reduction network are engineered to enhance the generator and mitigate noise and artifacts in images generated by GANs. A global local discriminator is used to enhance the overall image while adaptively adjusting the local region’s image effect. It addresses the problem of both excessive and insufficient enhancement in particular areas. Zhang et al.^63^ introduced an architecture termed as cascaded network with multi-level sub-networks (CNMS), which consists of the several essential elements: (a) a cascade framework using local modules and the global network using for obtaining feature representations with improved semantic depth and spatial precision, (b) information exchange among several resolution streams, and (c) a triple attention for the extraction of attention concentrated features. The CNMS model systematically integrates multiple sub-networks via triple attention modules for extracting the task-specific features from underwater images. This design enhances the network’s robustness and significantly improves its generalisation capability. In a related approach, Zhang et al.^64^ introduced a lightweight architecture called LiteEnhanceNet, specifically designed for single UIE. To reduce computational complexity, the network leverages depth wise separable convolutions as its core building blocks. Additionally, it incorporates a one-shot aggregation connection to efficiently capture features from both low level and intermediate layers. The key components such as carefully chosen activation functions and squeeze-and-excitation modules are strategically embedded to further optimize performance while maintaining a lightweight structure.

## Background

This section explains the concepts of underwater optimal imaging and examines the fundamentals of the morphological processed residuals system pertinent to the proposed method.

### Underwater image formation model

The Jaffe-McGlamey imaging framework^65^ proposed that underwater image formation results from the linear combination of direct, backscattered, and forward-scattered light components. The forward scattering effect is typically negligible. Consequently, the imaging model may be refined in the following manner:

1 $${I}_{c}= {J}_{c}{t}_{c}+ {A}_{c}\left(1-{t}_{c}\right), c \in \left\{R, G , B\right\},$$

In this context,$I$ represent the degraded underwater image, $J$ denotes the target object radiance for restoration, *A* signifies a global backscattered light and $t$ refers to the medium transmission map that relies on the objects distance and the water attenuation component, ${J}_{t}$ signifies forward scattering resulting in blurriness and chromatic aberrations, $A(1-t)$ represents the background scattering that mostly contributes to reduced contrast and color differences^66^. Both forward and backward scattering depends on the transmission map of the medium^67^. The application of a uniform attenuation coefficient in aquatic situations fails to accurately represent the phenomenon of selective light attenuation in water. Consequently, correcting wavelength dependent attenuation in underwater photographs is crucial for achieving enhanced quality results.

### Morphological processed residuals system

This section introduces a comprehensive description of the morphological processed residuals (MPR) system^68,69^

2 $$I=u\mathcal{*}\mathcal{L}$$

where * represents a convolutional function and $u$ signifies the input image. $\mathcal{L}$ indicates a Gaussian filter or any similar low pass filter mask. Thus, a linear filter residual is expressed as

3 $$Res\left(u\right)=u-I$$

Residual is further processed utilizing functions specified by images with positive values. In that situation, the *Res*(*u*), encompassing both positive and negative values is partitioned into two components: positive and negative.

4 $${I}_{res+}=0.5 \left(Res \left(u\right)+\left|Res \left(u\right)\right|\right), {I}_{res-}=0.5 \left(Res \left(u\right)-\left|Res \left(u\right)\right|\right)$$

The residual fraction exhibits the following seemingly connected relationship:

5 $$Res \left(u\right)= {I}_{res+}+ {I}_{res-}$$

Both components of the residual ${(I}_{res+}, {I}_{res-})$ are meticulously tuned to eliminate redundant inconsistencies while preserving relevant ones. The processing technique that identifies the most relevant residual segments while maintaining their original structures is introduced on the morphological function $\mathcal{M}$ and reconstruction. Lastly, significant residual segments are included into the image to enhance visible edges while the images continue to exhibit blurriness in insignificant locations.

6 $${I}_{out}=I+\mathcal{M}\left({I}_{res+}\right)-\mathcal{M}\left({I}_{res-}\right)$$

An operator $\mathcal{M}$, similar for both residuals is defined by

7 $$\mathcal{M}\left(I\right)= {R}_{I}\left(min\left(I, {S}_{t}\left(I\right){|}_{\left\{min\left\{I\right\}, max \left\{I\right\}\right\}}\right)\right)$$

where ${R}_{I}\left(A\right)$ denotes a morphological restoration of the grayscale mask image *I* employ the marker *A*, while ‘|’ denotes a mapping operator that reconstructs an image by replacing the binary values of 0 and 1 with two designated values. A ‘min’ denotes a pointwise minimum function applied to two images. A binary masker *S*, with key portions that preserve contrast, is essential for the analysis of $\mathcal{M}$. The replacement of these regions is determined by the extent of the residuals as,

8 $${S}_{t}\left(I\right)= \left(I \ge t\right)$$

where *S* denotes the selection function that identifies regions of $I$ with amplitudes surpassing a predetermined threshold $t$. The quantity of areas in which the initial sharpness is restored.

### Size criterion for selection of meaningful regions

The preliminary use of this technique demonstrates that the boundaries of relevant areas are defined in the binary mask obtained through thresholding. The significance is determined by the amplitude residual. At present, amplitude is not the sole determinant influencing the significance of an image component. The image coefficient may possess a higher residual amplitude specification without influencing image perception. The incorporation of salt and pepper into the image would produce multiple smaller components with increased amplitudes, modifying the original image. Consequently, they may be identified as pertinent components that are insufficient. Consequently, an auxiliary set is integrated into the original algorithm to address this issue. Binary mask is used for filtration by the area opening filter^69^. This filter eliminates components from the binary image that are smaller than the specified threshold $s$(size coefficient). Consequently, Eq. (8) is articulated as,

9 $${S}_{t,s}\left(I\right)= \left(I \ge t\right) o\left(s\right)$$

where $o(s)$ denotes an area opening that eliminates components less than the specified size $s$. However, it is evident that in both cases, as the component *s* in the modified approach and *t* in the original method increases, the number of selected regions reduces. However, in the first scenario, the application of coefficient *s* results in the elimination of components according to their magnitude. It allows for the elimination of smaller pixels and objects from residuals irrespective of their amplitudes being greater. Ultimately, it provides the ability to conceal these areas in the final image.

### Control contrast

The addition (or removal, depends upon the elements) of high pass filtering from the original image is a recognized method for enhancing image contrast. It relates to the high pass filtering attribute utilized to differentiate local variations in pixel values of an image. An alternative method to obtain high-pass filter outcomes is to examine the inconsistencies between low pass filtering and the original image. High frequency coefficient of images with amplitude regions surpassing threshold *t* are designated as morphologically processed residuals. A contrast control coefficient (*c*) is incorporated into Eq. (6) for adjusting the contrast in the final denoised image, yielding the revised formula as

10 $${I}_{out}=I+ \left(\mathcal{M}\left({I}_{res+}\right)-\mathcal{M}\left({I}_{res-}\right)\right).c.$$

The dependence on *c* could be further enhanced, maintained, and decreased. The impact of morphological processing on this information is evident in the abundance of significant image features^69^. This processing strategy enhances the contrast of relevant areas by eliminating extraneous information. This methodology is governed by three primary factors; *t*, *s*, and *c*. The quantity of information achieved depends on the residual magnitude (*t*) and the residual particle size (*s*). The parameter *c* enables for enhancement of the image contrast.

### Proposed methodology

**Fig. 1:**
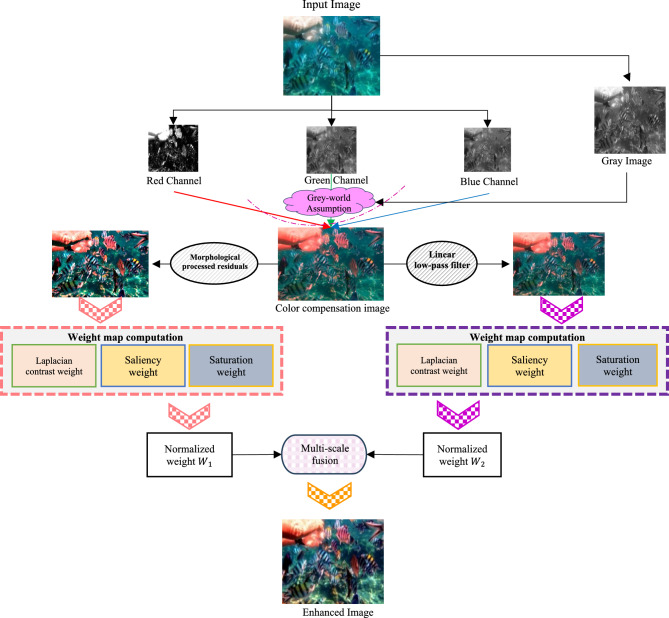
Methodological framework for the proposed UIE approach.

As illustrated in Fig. 1, the proposed image enhancement approach consists of a four-step process that includes color balancing, morphologically processed residuals, sharpening via a linear low-pass filter, and image fusion. Understanding the importance of color correction in underwater images, we propose to apply the color balance method to the raw input. This process removes unwanted color casts caused by varying light sources while enhancing overall image quality. Further, an effective fusion-based technique that utilizes morphologically processed residuals and sharpening to mitigate the haziness of the color-balanced image is used.

### Colour balancing

Color balancing primarily aims to correct unwanted color casts caused by differing lighting circumstances or medium attenuation qualities to improve the image’s quality^70^. The greenish-blue tint is a significant issue that necessitates solutions, as color perception in aquatic settings is intricately associated with depth. The vibrancy and appearance of a colorful surface are influenced by the selective attenuation of the wavelength spectrum when light traverses the water. In deeper water, the ability to perceive color reduces as long wavelengths are diluted more by scattering than short wavelengths. The overall distance between the observer and the scene affects both attenuation and chromatic deterioration in reality. Our analysis of the various modern color balancing techniques^71^ yielded several viable and context-specific solutions.

**Fig. 2:**
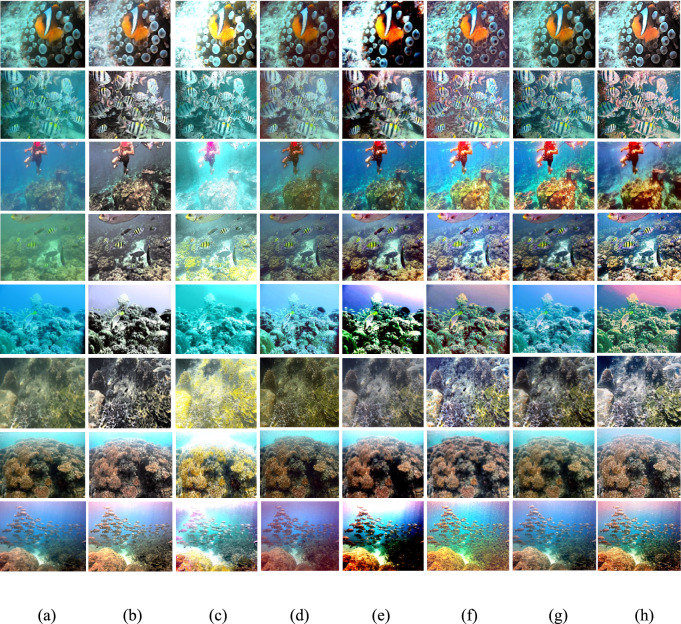
Comparison of intensity distributions for various enhancement methods: (**a**) Raw input, (**b**) CLAHE-PM, (**c**) JRL, (**d**) ARM, (**e**) IVP, (**f**) DRF, (**g**) DCP-DTP, (**h**) Proposed. The proposed approach achieves balanced color restoration and improved visual fidelity across different underwater background tones.

**Fig. 3:**
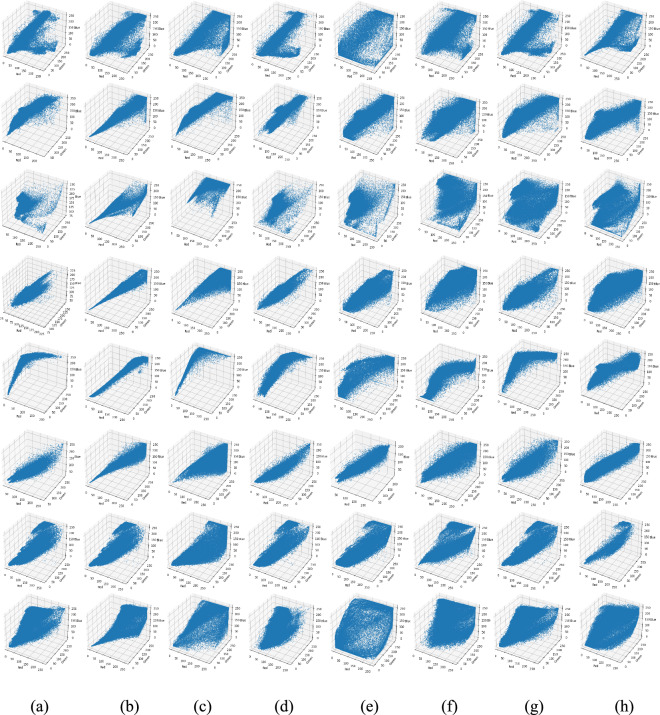
Intensity distributions corresponding to Fig. 2: (**a**) Raw input, (**b**) CLAHE-PM, (**c**) JRL, (**d**) ARM, (**e**) IVP, (**f**) DRF, (**g**) DCPDTP, (**h**) Proposed. Histogram representations of color in the proposed method exhibit a high uniform distribution across the entire spectrum, whereas the histograms of other methods exhibit concentration around specific values or intervals.

The Gray World method^72^ is recognized for producing exceptional visual results in somewhat distorted underwater environments. Nonetheless, extensive research demonstrates that most conventional approaches fail when applied to severely degraded underwater habitats. Their appearance is predominantly bluish owing to their incapacity to counteract the color shift. The Grey World approach displays significant red artifacts; yet, it is the most effective at removing the bluish color. Gray World approach normalizes each channel by its mean value, resulting in artifacts due to the red channel’s low mean value, which causes overcompensation in areas with high red intensity. To resolve this issue, we intend to rectify the inadequacy of the red channel, according with the findings of previous underwater research. We compute the color balanced image using the Gray World approach in a subsequent phase. To rectify the deficiencies of the red channel, we rely on the subsequent four observations; Green channel is significantly better preserved underwater compared to the red and blue channels. Red light, distinguished by its long wavelength is the first to be reduced while passing through clear water. (b) The green channel transmits opponent color information relative to the red channel, requiring modifications due to the greater attenuation of red in comparison to green. Thus, we mitigated red attenuation by incorporating a portion of the green channel into the red. Initially, we sought to incorporate both green and blue elements into the red; however, utilizing solely the green channel’s data facilitates a more effective restoration of the complete color spectrum while maintain the natural aesthetic of the background (aquatic areas). The compensation must align with the disparity between the mean green and mean red values as per the grey world assumption, which asserts that all channels possess equivalent mean values before attenuation; this difference signifies the imbalance in green and red attenuation. To prevent saturation of the red channel in the subsequent gray world phase following red loss compensation the enhancement of red can concentrate on pixels with reduced red channel values while maintain pixels with a substantial red component unchanged. Green channel data must not be conveyed in areas where the red channel data is substantial. Subsequently, our objective is to eliminate the reddish color produced by the Grey-world model in the overexposed regions. The modification of the red channel should be executed solely in regions exhibiting significant attenuation. This argument supports the assertion in^73^ that a pixel exhibiting substantial values across all three channels indicates its proximity to the observer or its position in a well-lit environment, thereby negating the necessity for restoration. Figures 2 and 3 illustrate the comparative intensity and histogram distributions of various enhancement methods, showing that the proposed approach achieves balanced color restoration, uniform intensity distribution, and improved visual fidelity across underwater scenes.

To address wavelength-dependent light attenuation and channel imbalance in underwater environments, adaptive compensation is applied to the red and blue channels before performing global color balancing. This step restores the lost color information due to differential absorption of light in water, particularly where the red and blue channels are significantly weakened. The compensation functions for the red and blue channels are defined as follows:

11 $${I}_{rc}\left(x\right)= {I}_{r}\left(x\right)+a. \left({\overline{I} }_{g}-{\overline{I} }_{r}\right). \left(1-{I}_{r}\left(x\right). {I}_{g}\left(x\right)\right)$$

12 $${I}_{bc}\left(x\right)= {I}_{b}\left(x\right)+a. \left({\overline{I} }_{g}-{\overline{I} }_{b}\right). \left(1-{I}_{b}\left(x\right). {I}_{g }\left(x\right)\right)$$

In Eqs. (11) and (12), ${I}_{rc}(x)$ and ${I}_{bc}(x)$ denote the compensated red and blue channel intensities at the pixel position $x$, respectively. ${I}_{r}(x)$, ${I}_{g}(x)$, and ${I}_{b}(x)$ represent the normalized intensity values (ranging from 0 to 1) of the red, green, and blue channels of the input image $I$. The parameters ${\bar{I}_{r}}$, ${\bar{I}_{g}}$, and ${\bar{I}_{b}}$ indicate the mean intensity values of the respective channels computed over the entire image, which describe the global attenuation level of each spectral component. The constant $\alpha$ is a color compensation coefficient, empirically set to 1.0, that regulates the strength of the correction. The terms $({\bar{I}_{g}}-{\bar{I}_{r}})$ and $({\bar{I}_{g}}-{\bar{I}_{b}})$ measure the attenuation difference between the green channel and the red or blue channels, respectively, using the green channel as a reference because it experiences the least absorption in underwater environments^74^. The nonlinear adaptive term $(1-{I}_{r}(x)\cdot {I}_{g}(x))$ or $(1-{I}_{b}(x)\cdot {I}_{g}(x))$ ensures spatial adaptivity by applying greater compensation to pixels with low red or blue intensities while avoiding over-enhancement in regions that are already bright. This adaptive correction mechanism effectively balances color restoration across varying depths and illumination conditions, ensuring natural visual appearance and minimizing oversaturation. Following the red and blue channel compensation, the standard Gray World assumption is applied to normalize the global color distribution, resulting in an overall color-balanced underwater image with improved perceptual quality.

### Multiscale fusion

This research used multiscale fusion approach to create a way for enhancing underwater photographs. Image fusion has demonstrated efficiency in various applications including medical imaging^75,76^, multispectral video enhancement^77^, dehazing^70^, and high dynamic range (HDR) imaging^78^. Our objective is to develop a simple and effective approach that enhances scene visibility in several underwater observations. Our methodology is based on a synthesis of inputs and weights maps obtained from a singular source image. Nonetheless, these are carefully selected to improve the color-balancing technique. Two inputs are utilized for enhancing color contrast an edge sharpness of the color balanced image while weight maps are generated to maintain qualities and repair the deficiencies of those inputs, thereby alleviating artifacts caused by light propagation limitations in underwater environments. Previous methodologies indicated that the backscattering component resulting from artificial light interacting with water particles and reflecting back to the camera has a reduced effect on the data derived from the original image. This assumption is typically applicable in underwater habitats adequately lighted by natural light, but it is ineffective under more demanding lighting conditions. This study ignores the optical model and proposes an alternative framework for inputs and weights to address significantly degraded situations.

### Inputs of the fusion process

**Fig. 4:**
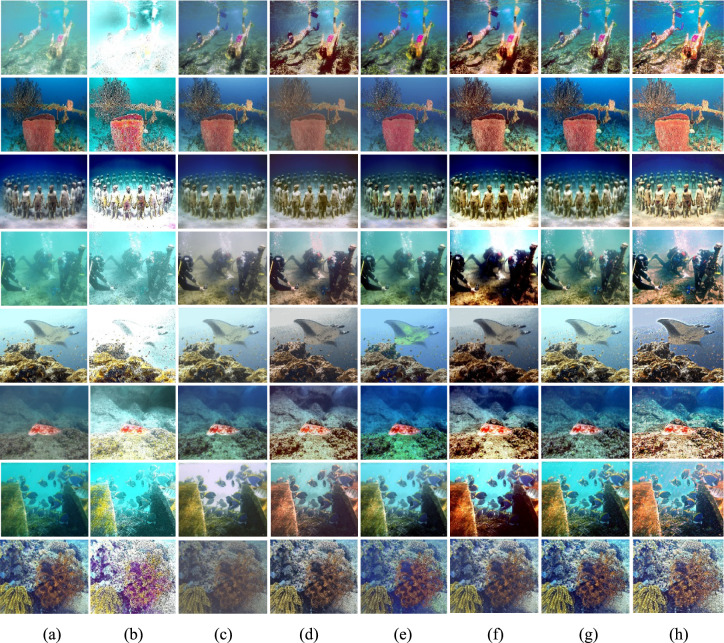
Comarison of color correction: (**a**) Raw image, (**b**) CLAHE-PM, (**c**) JRL, (**d**) ARM, (**e**) IVP, (**f**) DRF, (**g**) DCP-DTP, (**h**) Proposed. The proposed technique effectively restores natural color tones and contrast, eliminating color casts prevalent in other approaches.

**Fig. 5:**
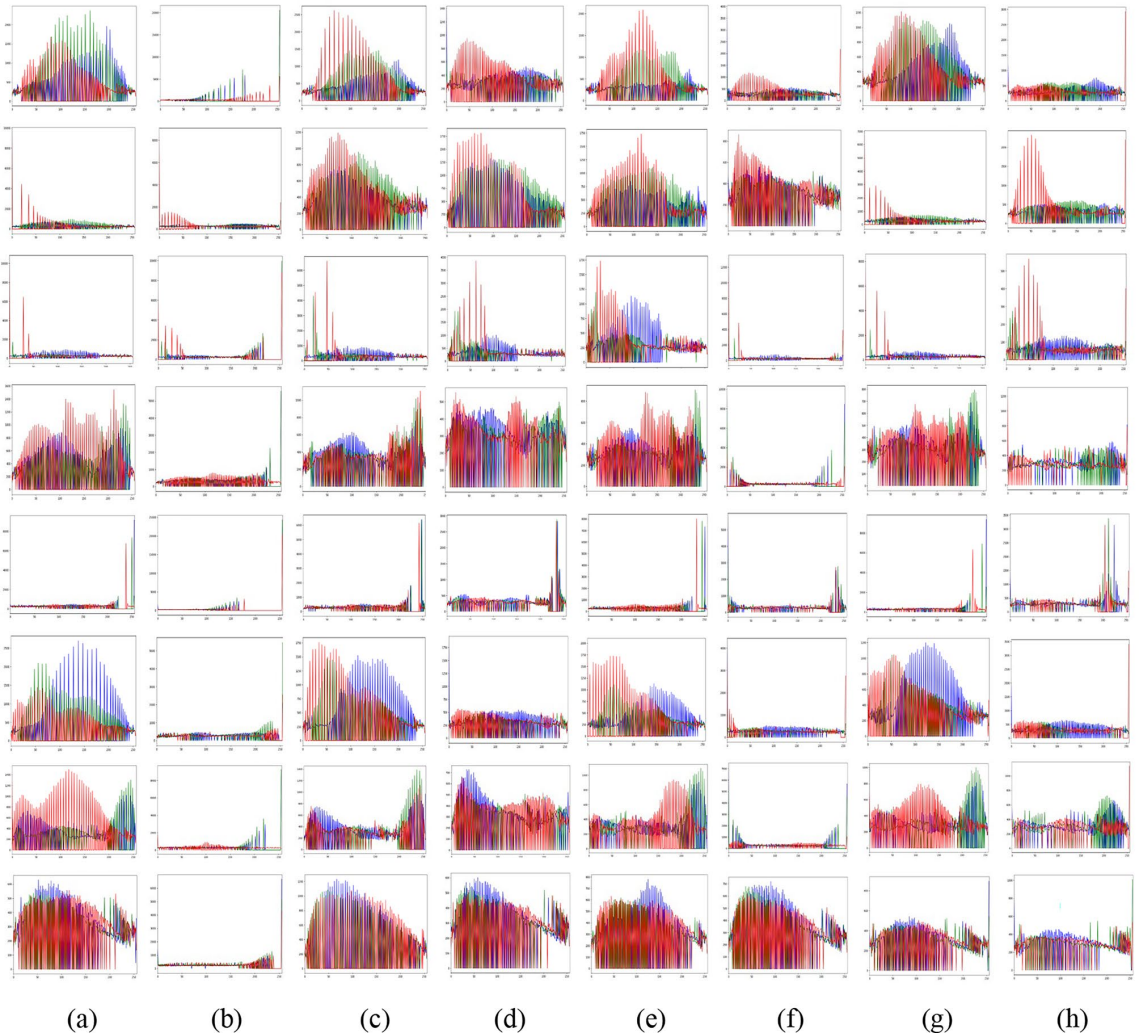
Color histogram distributions corresponding to Fig. 4. (**a**) Raw input, (**b**) CLAHE-PM, (**c**) JRL, (**d**) ARM, (**e**) IVP, (**f**) DRF, (**g**) DCP-DTP, (**h**) Proposed method. The proposed method produces uniform spectral balance, demonstrating accurate color correction and reduced over-saturation artifacts.

Given the significance of color correction in underwater environments, we initially use our color balance technique on the original image. The stage aims to improve image quality by eliminating undesirable color casts produced by different light sources. In water over 30 feet in depth, color balancing is adversely affected due to challenges in retrieving absorbed colors. Consequently, to acquire our first input, we employ an edge-preserving method for color images known as morphologically processed residuals (MPR). It integrates linear low pass filtering with nonlinear methods, enabling the identification of important image regions where edge preservation is crucial. The identification of these regions rely on the morphological examination of linear filter residuals, with the objective of pinpointing significant areas defined by prominent edges of substantial amplitude and suitable dimensions. Two approaches of morphological image processing are employed for their identification: (a) the reconstruction function and area opening. The significant recreated areas are then integrated with the low pass filtering outcomes to restore the original edge outlines. Furthermore, the technology facilitates the alteration of the output image’s contrast. The outcome of the processing depends upon four elements, the selection of which enables the modification of the processed image to meet specific criteria. We obtain an additional input that aligns with an enhanced version of the color-balanced image. Consequently, we employ the unsharp masking technique by merging a blurred or unsharp (specifically Gaussian-filtered) variant of the image with the original to enhance sharpness. The typical equation for unsharp masking describes the sharpened image $S$, as $S= \left(I+\beta \left(I-G*I\right)\right)$, where $\mathrm{I}$ represents an image to be sharpened (specifically, a color-balanced image), $G*I$ is the gaussian filtered versions of $I$ and $\beta$ is the parameter. The determination of *β* is not uncomplicated in practice. A negligible *β* does not enhance $I$, while an excessively large $\beta$ leads to over-saturation characterized by prominent highlights and excessive shadows. To address this issue, we define an enhancement image *S* as follows:

13 $$S=\frac{\left(I+ \mathcal{N}\left\{I-G*I\right\}\right)}{2},$$

where $N \{.\}$ represents a linear normalizing function, commonly referred as histogram stretching in academic literature. This function adjusts and scales the intensity of each color pixel in an image using specified shifting and scaling factors, ensuring that the modified pixel values encompass the whole available dynamic range. The sharpening technique will henceforth be termed the normalized unsharp masking process. It provides the advantage of necessitating no parameter adjustments and demonstrates efficiency in sharpening. This additional input markedly mitigates the degradation resulting from scattering. The difference between a color-balanced image and its Gaussian filtered counterpart signifies a high pass signal similar to the inverse of the Laplacian. This approach frequently intensifies high frequency noise and causes undesired distortions in the secondary input^79^. Multiscale fusion technique outlined in the subsequent section will alleviate the transfer of artifacts to the final composite image. Figures 4 and 5 show that the proposed method restores natural color tones with uniform spectral balance and minimizes over-saturation compared to other approaches.

### Weights computation for fusion process

In image fusion, weight maps are employed to ensure that pixels with higher weights contribute more significantly to the final output. These weights are typically derived from various local image quality or saliency features. For example, the Laplacian contrast weight ${W}_{L}$ captures global contrast by computing the absolute response of a Laplacian filter applied to the luminance channel of each input image This specific indicator is utilized in several applications including tone mapping^78^ and enhancement of depth of focus^80^ as it allocates higher values to edges and textures. The weight utilized in the underwater dehazing process is inadequate for contrast restoration, mostly due to its ineffectiveness in differentiating between ramp and flat regions. We proposed an extra supplementary metric for contrast assessment to address this issue. The saliency weight ${W}_{S}$ is designed to highlight salient objects that reduce in importance within the undersea environment. We utilized the saliency estimator developed by Achantay et al.^81^ for determining the level of saliency. This computationally efficient technique is derived from the biological principle of center surround contrast. However, the saliency map typically accentuates illuminated regions (areas with increased luminance values). To address this issue, we proposed a supplementary weight map derived from the finding that saturation reduces in the focused regions. The saturation weight ${W}_{Sat}$ enables the fusion method for adapting to chromatic information by giving greater emphasis to highly saturated regions in the image. For each input image ${I}_{k}$, the saturation weight map is computed by measuring the deviation of the red (${R}_{k}$), green (${G}_{k}$), and blue (${B}_{k}$) channels from the corresponding luminance (${L}_{k}$) at each pixel location. This highlights areas with rich color content, contributing to a more visually appealing fused result.

14 $${W}_{Sat}=\sqrt{1/3\left[{\left({R}_{k}-{L}_{k}\right)}^{2}+ {\left({G}_{k}-{L}_{k}\right)}^{2}+{\left({B}_{k}-{L}_{k}\right)}^{2}\right]}$$

In practice, the three individual weight maps including Laplacian contrast ${W}_{L}$, saliency ${W}_{S}$, and saturation ${W}_{Sat}$ are combined to form a single aggregated weight map for each input image. Specifically, for each input *k*, the aggregated weight map ${W}_{k}$​ is obtained by summing the respective weight maps. These $K$ aggregated maps are then normalised on a pixel-by-pixel basis, where the weight of each pixel is divided by the sum of corresponding pixel values across all input maps. This process yields the normalized weight maps ${\overline{W} }_{k}$ for each input, ensuring that the fusion process preserves balanced contributions from all inputs.

15 $${\overline{W} }_{k}=\frac{\left({W}_{k}+\delta \right)}{\left({\sum }_{k=1}^{K}{W}_{k}+K.\delta \right),}$$

where *δ* is an insignificant regularization term that guarantees the influence of each input on the output. *δ* is established at 0.1 for the entirety of the trial. In contrast to prior studies^77^, we restrict our analysis to these three weight maps and do not calculate the exposedness weight maps. While the use of only two input images, as proposed in this study, helps reduce the overall complexity of the fusion process, we observed certain trade-offs in visual quality. Specifically, although one input effectively enhances edge features such as the ramp edges in the second input; the contrast benefits offered by the gamma-corrected image tend to reduce. This can be explained as follows: (a) In the context of exposure fusion^78^, the exposedness weight map is designed to down weight pixels that are either under-exposed or over-exposed. As a result, it assigns higher weights to pixels near the center of the image’s dynamic range and lower weights to those at the extremes. Since the gamma-corrected image spans the full dynamic range, it is penalized by the exposedness weight map. In contrast, the sharper input favoured by this map contributes disproportionately, leading to sharpening artifacts and suboptimal contrast enhancement in the final fused output.

### Fusion process

A reconstructed image $\mathcal{P}\left(x\right)$ is generally derived by integrating the chosen inputs with the weight measures at each pixel location (*x*) in accordance with the normalized weight maps.

16 $$\mathcal{P}\left(x\right)= {\sum }_{k=1}^{K}{\overline{W} }_{k} \left(x\right) {I}_{k}(x)$$

In this case, ${I}_{k}$ represents an input, where *k* indicates an index of the inputs *K* = 2, which is multiplied by the normalised weight mapping ${\overline{W} }_{k}$. The rudimentary method, in fact, yields unwanted halos^77^. A prevalent remedy to mitigate this constraint is the application of multiscale linear^82^ or nonlinear filters^83^.

#### Multiscale fusion process

The multi-scale decomposition employed in this work is based on the Laplacian pyramid originally introduced by Burt and Adelson^80^. Pyramid representation breaks down an image into a series of band-pass images that capture details at different spatial frequencies. At each pyramid level, a low Gaussian filter *G* is applied to the input image, reducing its resolution by the factor of 2 in both spatial dimensions. The resulting low pass image is then upsampled and subtracted from the original input, yielding a band-pass image that approximate the inverse Laplacian. The decimated low pass image is then utilised as input for the next pyramid level. Formally, the *N* levels ${L}_{l}$ of the Laplacian pyramid are constructed using a sequence of $l$ low pass filtering and downsampling operations, denoted by ${G}_{l}$​, followed by $l$ upsampling steps to reconstruct the band-pass components at each level.

17 $$\begin{aligned} I\left( x \right) & = I\left( x \right) - G_{1} \left\{ {I\left( x \right)} \right\} + G_{1} \left\{ {I\left( x \right)} \right\} \\ & \triangleq L_{1} \left\{ {I\left( x \right)} \right\} + G_{1} \left\{ {I\left( x \right)} \right\} \\ & = L_{1} \left\{ {I\left( x \right)} \right\} + G_{1} \left\{ {I\left( x \right)} \right\} - G_{2} \left\{ {I\left( x \right)} \right\} + G_{2} \left\{ {I\left( x \right)} \right\} \\ & = L_{1} \left\{ {I\left( x \right)} \right\} + L_{2} \left\{ {I\left( x \right)} \right\} + G_{2} \left\{ {I\left( x \right)} \right\} \\ & = _{{......}} \\ & = \sum _{{l = 1}}^{N} L_{l} \left\{ {I\left( x \right)} \right\} \\ \end{aligned}$$

In the above formulation, ${L}_{1}$ and ${G}_{1}$ represent the ${l}\mathrm{th}$ levels of the Laplacian and Gaussian pyramids, respectively. For equation consistency, all images are assumed to be upsampled to match the original image dimensions. However, in practical implementations, each pyramid level is processed at its native subsampled resolution for high computational efficiency. Following the standard multiscale fusion framework^78^, each source image ${I}_{k}$ is decomposed into a Laplacian pyramid^80^, while the corresponding normalised weight maps ${\overline{W} }_{k}$ are decomposed using a Gaussian pyramid. Both pyramids share the same pixel numbers and the fusion process occurs independently at each level $l$ by combining the Laplacian components of the source images with the Gaussian-weighted maps.

18 $${\mathcal{P}}_{l}\left(x\right)= {\sum }_{k=1}^{K}{G}_{l}\left\{{\overline{W} }_{k}(x)\right\} {L}_{l}\left\{{I}_{k}(x)\right\}$$

where $l$ is the pyramid levels and *k* signify the quantity of input photographs. The quantity of levels *N* is relied on the image dimensions and directly influences the perceptual quality of the composite image. The enhanced output is derived by combining the integrated contributions from all levels following suitable up-sampling. The independent use of a fusion technique at each scale level alleviates artifacts resulting from abrupt changes in the weight maps. Multiscale fusion is inspired by the human visual system where it demonstrates increased sensitivity to abrupt changes in smooth image patterns while interpreting significant reduced sensitivity to irregularities observed at edges and textures. The study demonstrated that the multiscale procedure could be effectively emulated by a computationally efficient and aesthetically pleasing single scale system^84^.

### Parameter selection and justification

The performance of the proposed UIE framework largely depends on three key parameters namely, the morphological amplitude threshold (*t*), the region size threshold (*s*), and the contrast control coefficient (*c*) along with the fusion weight selection used during multiscale image fusion. Each of these parameters influences the overall enhancement quality, structural clarity, and color balance of the restored image. The amplitude threshold (*t*) defines the minimum residual magnitude that is preserved during morphological reconstruction. This parameter ensures that fine image structures are retained while insignificant or noisy residuals are suppressed. Experimental trials demonstrated that smaller threshold values (below 0.001) tend to retain excessive fine details but also amplify background noise, whereas larger thresholds (above 0.005) result in the loss of subtle edges and fine textural information. Based on empirical optimization across multiple benchmark datasets, a value of *t* = 0.001 was found to offer the best compromise between edge preservation and noise suppression.

Similarly, the region size threshold (*s*) governs the minimum connected area of residual components that are maintained in the reconstructed image. When *s* is set too low (less than 0.05), random pixel fluctuations and isolated noise artifacts are preserved, leading to structural inconsistency. Conversely, higher values (greater than 0.2) remove smaller edge regions and cause over smoothing. The optimal selection of *s* = 0.1 was determined through perceptual evaluation and quantitative measures such as local entropy and edge sharpness, ensuring accurate boundary retention without amplification of noise. The contrast control coefficient (*c*) in the luminance adjustment equation regulates the strength of contrast amplification. Lower values (< 1.5) were observed to produce insufficient enhancement, resulting in underexposed images, while excessively high values (> 3.0) generated over-saturated or halo artifacts around high-intensity regions. Through a series of quantitative evaluations using peak signal-to-noise ratio (PSNR), structural similarity index measure (SSIM), underwater image quality measure (UIQM) metrics, a coefficient value of *c* = 2.4 yielded the highest overall enhancement consistency and perceptual quality. This value ensures natural contrast improvement and effective detail enhancement without compromising color fidelity.

During multiscale fusion, three weight components; Laplacian contrast (WL), saliency (WS), and saturation (WSAT) were adaptively computed to control the contribution of structural, contrast, and chromatic information. The selection of these weights follows the principle of visual attention theory, in which the human visual system gives priority to edges and color salient regions. The fusion process normalizes these weight maps to maintain a balanced contribution between structural enhancement and color naturalness. Experimental observation indicated that increasing WL sharpens structural details but may slightly reduce color harmony, while higher WSAT improves color richness but can lower contrast uniformity. The adopted normalization strategy automatically balances these effects, leading to perceptually optimal image fusion across all tested datasets.

### Sensitivity analysis

To further justify the reliability of the selected parameters, a comprehensive sensitivity analysis was conducted by varying the values of *t*, *s*, and *c* within their practical ranges. The impact of each parameter variation on the enhancement quality was assessed using standard quantitative metrics, including PSNR, SSIM, UIQM, and underwater color image quality evaluation (UCIQE), across multiple test images. The analysis revealed that minor deviations (± 10%) from the optimal values produced negligible changes in the output quality, confirming the stability and robustness of the proposed configuration. Specifically, PSNR and SSIM values remained nearly constant within 0.2–0.3 dB and 0.003–0.005 variations, respectively, while perceptual metrics such as UIQM and UCIQE showed consistent trends. These results indicate that the chosen parameters not only provide optimal visual enhancement but also maintain consistent performance across diverse underwater scenes and lighting conditions.

**Fig. 6:**
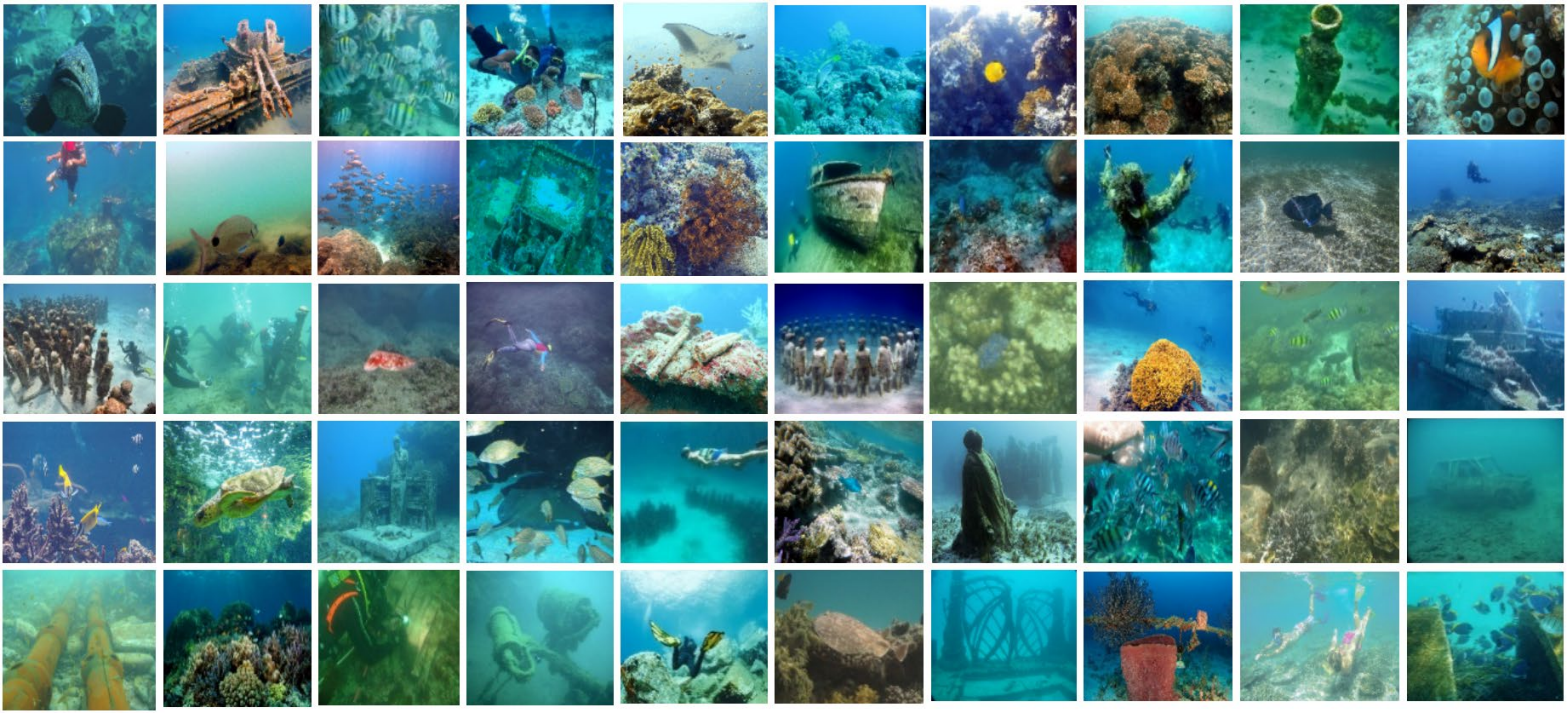
Representative underwater images used for testing, showing varied turbidity levels, lighting, and background colors in the evaluation datasets.

Furthermore, the sensitivity evaluation highlighted that the proposed method demonstrates low dependency on parameter tuning, offering high generalization capability and practical adaptability for real-time underwater imaging applications. The morphological thresholds effectively preserve meaningful structural components without requiring image-specific adjustment, and the contrast control coefficient ensures perceptual uniformity across scenes with varying illumination intensities. The robustness of the parameter selection thus validates the technical soundness and reproducibility of the proposed enhancement framework, confirming its applicability in both controlled and in situ underwater imaging conditions. Figure 6 presents representative underwater test images depicting varied turbidity, lighting, and background conditions used for evaluation.

## Results and discussion

For evaluating the overall performance of the proposed method, experiments were conducted using underwater images from two standard benchmark dataset i.e. underwater image enhancement benchmark (UIEB)^73,85–87^ and the large-scale underwater image (LSUI) dataset^56^. The UIEB dataset contains 950 real-world underwater images, including 890 pairs with perceptually selected reference images and 60 challenging unpaired samples, covering various degradation levels caused by light scattering and color attenuation. The LSUI dataset comprises 4,279 underwater image pairs with corresponding reference, semantic, and transmission maps, representing diverse underwater scenes under varying illumination and turbidity conditions. These datasets were chosen due to their comprehensiveness and wide adoption in recent underwater enhancement studies, ensuring a fair and standardized comparison. Representative images were selected from the UIEB and LSUI datasets for evaluation to ensure diverse environmental coverage and consistent benchmarking across all comparative enhancement methods. All the experiments are conducted on a personal computer with an Intel Core i7-4790 CPU (3.60 GHz), 8 GB of RAM, and a 64-bit Windows 10 operating system in MATLAB R2021a as the main computational platform. In the experiments, key parameters including the amplitude threshold (*t*), threshold size (*s*), contrast coefficient (*c*), and sigma of the low pass filter are empirically set to 0.001, 0.1, 2.4, and 15, respectively. These values effectively balance algorithm convergence and computational efficiency while ensuring satisfactory enhancement results. The empirical parameter configurations yield optimal performance across a wide range of underwater images. A detailed analysis of parameter selection and its impact on image enhancement is further explored in the subsequent parameter assessment experiment. For a fair and reproducible comparison, the quantitative scores of recent deep-learning-based methods were adopted from their respective published papers when trained and tested on the same benchmark datasets (UIEB, LSUI). For methods with publicly available pretrained models, the results were verified by reproducing their outputs under identical test conditions and parameter settings. The proposed method, being non-learning-based was evaluated on the same datasets using identical experimental parameters to ensure consistent and equitable comparison.

### Performance evaluation metrics

For illustrating efficiency of the proposed method, our method is compared with twenty-two recent UIE techniques, including contrast-limited adaptive histogram equalization (CLAHE) and percentile methodologies (CLAHE-PM)^24^, joint residual learning (JRL)^25^, adaptive retinal mechanisms (ARM)^27^, improved visual perception (IVP)^26^, deep residual network (DRF)^29^, DCP and depth transmission map (DCP-DTP)^28^, depth map and illumination estimation (DMIE)^14^, joint luminance and chrominance learning (JLCL)^67^, two-step domain adaptation (TSDA)^60^, Bayesian retinex (Bret)^85^, light scattering characteristics (LSC)^35^, reinforcement learning (ReinL)^57^, adaptive learning attention network (ALANet)^61^, deep transfer learning base on a colour restoration model (DTL-CRM)^18^, adaptive colour correction and improved retinex algorithm (ACC-IRA)^36^, embedded fusion mechanism (EFM)^34^, cascaded multi-level networks and triple attention mechanism (CMS-TAM)^63^, unpaired image to -image translation (UMGAN)^62^, dual attention transformer (UDAformer)^54^, hybrid fusion method (HFM)^41^, U-shape transformer (USTrans)^56^, a lightweight network (LiteEnhanceNet)^64^. The performance of our method along with other competing methods are evaluated using the following quantitative metrics:

- **UIQM:** The underwater image quality measure^85^ incorporate three different metrics: UISM for sharpness, UIConM for contrast, and UICM for colourfulness. Higher UIQM values indicate superior enhancement through a balanced improvement in all three aspects.
- **UCIQE:** Underwater color image quality^85^ is evaluated using a linear combination of chroma, saturation and the contrast capturing the effects of non-uniform color casts, image blur, and low contrast. Higher UCIQE scores signify enhanced equilibrium among chroma, saturation, and contrast within the image.
- **PSNR**: PSNR^88^ is a standard metric that evaluates the quality of a reconstructed or compressed image compared to the original image. It is utilized to evaluate the degree of distortion or noise created during compression, denoising, or other image processing techniques. A higher PSNR indicates enhanced image quality (decreased distortion), while a lower PSNR represents reduced image quality (increased distortion).
- **SSIM**: Structural similarity^88^ is a commonly used tool for evaluating the image quality. While PSNR emphasizes pixel differences, SSIM captures variations in structural information, luminance, and contrast, offering a representation of image quality that aligns more closely with human visual perception. SSIM ranges from − 1 to 1, where 1 indicates perfect structural similarity between two images, 0 denotes a lack of structural similarity, and − 1 signifies complete dissimilarity.
- **Entropy**: Entropy^85^ indicates the average information contained in an image with a higher Entropy value denoting a greater quantity of information in the image.
- **PIQE**: The perceptual image quality evaluator^47^ is a non-reference image quality assessment (IQA) metric. PIQE examines the quality of an image independently of the input. The objective is to predict the perceptual quality of an image by assessing various distortion types while making it advantageous in scenarios where a reference image is unavailable. The PIQE score spans from 0 to 100, where lower values indicate higher image quality and larger values reflect lower image quality.

**Note:** In this study, PSNR and SSIM were computed using the paired subset of the UIEB dataset, where each degraded underwater image is accompanied by a perceptually selected reference image rather than a physically captured ground truth. These reference images serve as proxy ground truths for quantitative evaluation, acknowledging the inherent limitations of real underwater datasets. Table 1 summarizes of comparative UIE methods and their abbreviations.

**Table 1:** Summary of comparative UIE methods and their abbreviations.

Acronym	Full name/method	Core concept/Description
CLAHE-PM	Contrast limited adaptive histogram equalization with percentile method	Enhances local contrast by adaptive histogram equalization and percentile intensity mapping to prevent over-amplification
JRL	Joint residual learning	Deep learning framework combining residual learning for illumination and structure recovery
ARM	Adaptive retinal mechanism	Mimics human retinal adaptation to enhance contrast and local luminance in underwater images
IVP	Improved visual perception	Statistical enhancement method improving perceptual visibility through adaptive intensity correction
DRF	Deep residual framework	CNN-based enhancement using residual blocks to suppress haze and improve fine details
DCP-DTP	Dark channel prior with depth transmission map	Model-based dehazing using transmission estimation from dark channel statistics
DMIE	Depth map and illumination estimation	Employs scene depth and illumination priors to restore color and reduce backscattering
JLCL	Joint luminance and chrominance learning	Dual-branch network that jointly learns brightness and color information for enhancement
TSDA	Two-step domain adaptation	Domain adaptation method improving generalization between synthetic and real underwater domains
BRet	Bayesian retinex	Uses Bayesian inference within Retinex theory to recover illumination and reflectance for color constancy
LSC	Light scattering characteristics	Physically inspired model estimating light attenuation for improved visibility
ReinL	Reinforcement Learning Framework	Learns optimal enhancement actions as sequential decisions through reward-based training
ALANet	Adaptive Learning Attention Network	Deep supervised attention model integrating pixel and channel attention for adaptive enhancement
DTL-CRM	Deep transfer learning based color restoration model	Transfer learning-based model performing domain transformation and color restoration
ACC-IRA	Adaptive color correction and improved retinex algorithm	Combines adaptive color compensation with Retinex-based contrast and illumination adjustment
EFM	Embedded fusion mechanism	Multi-input fusion framework using contrast and saliency weight maps for refined enhancement
CMS-TAM	Cascaded multi-level network with triple attention mechanism	Hierarchical deep network employing triple attention for detail preservation and color fidelity
UMGAN	Underwater multiscale generative adversarial network	GAN-based enhancement model for unpaired underwater–in-air image translation
UDAformer	Unpaired dual attention transformer	Transformer-based framework integrating dual attention for structure and color enhancement
HFM	Hybrid fusion method	Combines color balance, fuzzy visibility recovery, and nonlinear mapping for improved fusion
USTrans	U-shape transformer network	Encoder-decoder transformer capturing global and local context for underwater image correction
LiteEnhanceNet	Lightweight enhancement network	Compact CNN using depthwise separable convolutions for fast and efficient enhancement

### Qualitative evaluation

**Fig. 7:**
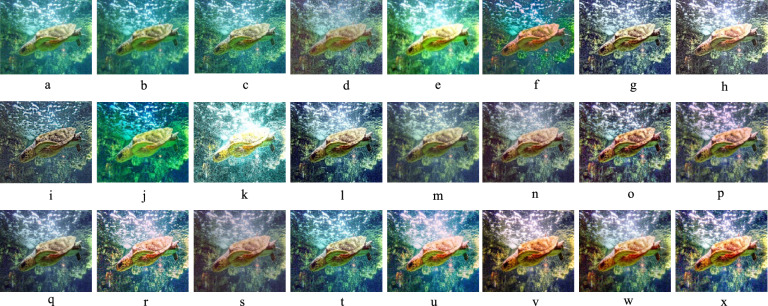
Qualitative enhancement results for the *Turtle* image: (**a**) Source image, (**b**) CLAHE-PM, (**c**) JRL, (**d**) ARM, (**e**) IVP, (**f**) DRF, (**g**) DCP-DTP, (**h**) DMIE, (**i**) JLCL, (**j**) TSDA, (**k**) BRet, (**l**) LSC, (**m**) ReinL, (**n**) ALANet, (**o**) DTL-CRM, (**p**) ACC-IRA, (**q**) EFM, (**r**) CMS-TAM, (**s**) UMGAN, (**t**) UDAformer, (**u**) HFM, (**v**) USTrans, (**w**) LiteEnhanceNet, (**x**) Proposed (Zoom in for enhanced detail visibility across all images).

**Fig. 8:**
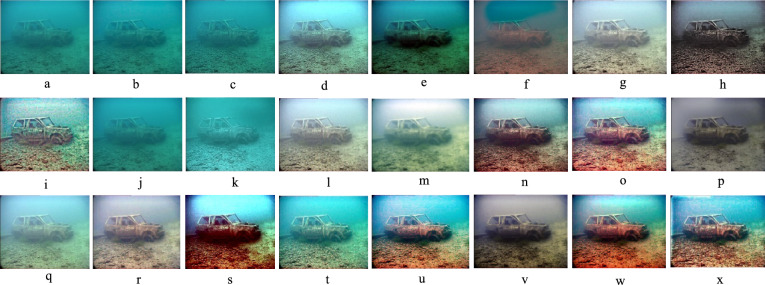
Qualitative enhancement results for the *Car* image: (**a**) Source image, (**b**) CLAHE-PM, (**c**) JRL, (**d**) ARM, (**e**) IVP, (**f**) DRF, (**g**) DCP-DTP, (**h**) DMIE, (**i**) JLCL, (**j**) TSDA, (**k**) BRet, (**l**) LSC, (**m**) ReinL, (**n**) ALANet, (**o**) DTL-CRM, (**p**) ACC-IRA, (**q**) EFM, (**r**) CMS-TAM, (**s**) UMGAN, (**t**) UDAformer, (**u**) HFM, (**v**) USTrans, (**w**) LiteEnhanceNet, (**x**) Proposed (Zoom in for enhanced detail visibility across all images).

**Fig. 9:**
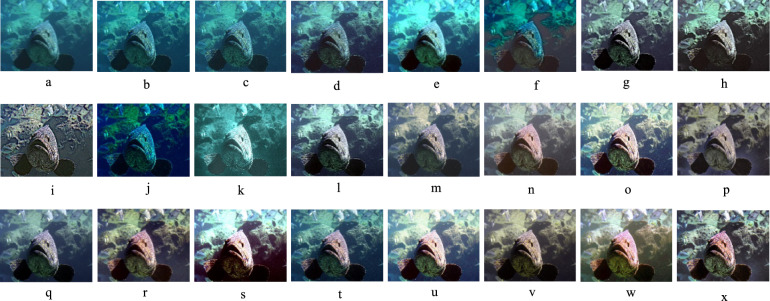
Qualitative enhancement results for the *Fish* image. (**a**) Source image, (**b**) CLAHE-PM, (**c**) JRL, (**d**) ARM, (**e**) IVP, (**f**) DRF, (**g**) DCP-DTP, (**h**) DMIE, (**i**) JLCL, (**j**) TSDA, (**k**) BRet, (**l**) LSC, (**m**) ReinL, (**n**) ALANet, (**o**) DTL-CRM, (**p**) ACC-IRA, (**q**) EFM, (**r**) CMS-TAM, (**s**) UMGAN, (**t**) UDAformer, (**u**) HFM, (**v**) USTrans, (**w**) LiteEnhanceNet, (**x**) Proposed (Zoom in for enhanced detail visibility across all images).

**Fig. 10:**
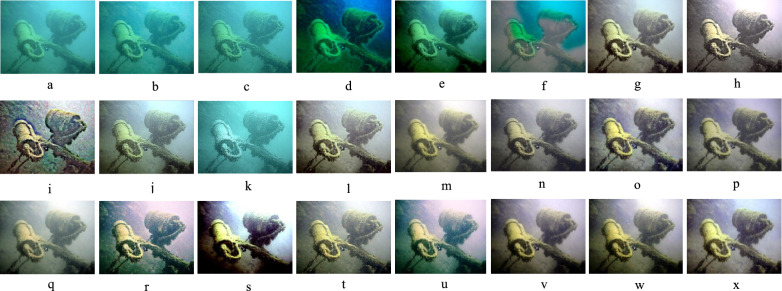
Qualitative enhancement results for the *Photographic* image: (**a**) Source image, (**b**) CLAHE-PM, (**c**) JRL, (**d**) ARM, (**e**) IVP, (**f**) DRF, (**g**) DCP-DTP, (**h**) DMIE, (**i**) JLCL, (**j**) TSDA, (**k**) BRet, (**l**) LSC, (**m**) ReinL, (**n**) ALANet, (**o**) DTL-CRM, (**p**) ACC-IRA, (**q**) EFM, (**r**) CMS-TAM, (**s**) UMGAN, (**t**) UDAformer, (**u**) HFM, (**v**) USTrans, (**w**) LiteEnhanceNet, (**x**) Proposed (Zoom in for enhanced detail visibility across all images).

**Fig. 11:**
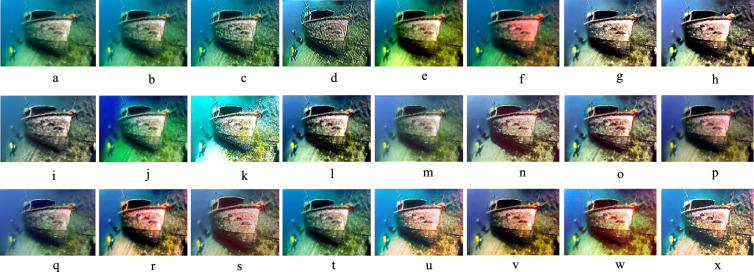
Qualitative enhancement results for the *Ship* image: (**a**) Source image, (**b**) CLAHE-PM, (**c**) JRL, (**d**) ARM, (**e**) IVP, (**f**) DRF, (**g**) DCP-DTP, (**h**) DMIE, (**i**) JLCL, (**j**) TSDA, (**k**) BRet, (**l**) LSC, (**m**) ReinL, (**n**) ALANet, (**o**) DTL-CRM, (**p**) ACC-IRA, (**q**) EFM, (**r**) CMS-TAM, (**s**) UMGAN, (**t**) UDAformer, (**u**) HFM, (**v**) USTrans, (**w**) LiteEnhanceNet, (**x**) Proposed (Zoom in for enhanced detail visibility across all images).

The proposed method is evaluated against 22 state-of-the-art UIE techniques using a dataset comprising over 50 raw underwater photographs. Representative results from diverse underwater scenes are illustrated in Figs. 7, 8, 9, 10, and 11 (UIEB dataset), and Figs. 13 and 14 (LSUI dataset) to highlight the comparative performance of these methods. In Figs. 7, 8, 9, 10 and 11, methods such as CLAHE-PM, JRL, ARM, IVP, DRF, and DCP-DTP exhibit limited effectiveness in removing color casts and enhancing contrast. This is particularly evident in their inability to address the non-uniform illumination and color distortions commonly found in underwater environments. Notably, the application of the color constancy hypothesis suitable for natural image enhancement proves inadequate in underwater settings, as seen in JRL and DRF, where excessive red channel compensation results in unnatural color shifts. Methods like DMIE and JLCL also struggle due to inaccurate light modelling, leading to suboptimal color correction and structural enhancement. This is visually apparent in Figs. 7, 8, 9, 10 and 11h, where processed images exhibit strong red dominance and overall darkness. ReinL demonstrates improved performance by employing a robust color correction strategy coupled with a variational Retinex-based framework to enhance structural details. However, its reliance on first-order gradients visible in Figs. 7, 8, 9, 10 and 11m introduces blurring and excessive enhancement artifacts. While TSDA shows noticeable contrast improvement in Figs. 7, 8, 9 and 10j, its performance deteriorates under severely degraded conditions, as seen in residual artifacts (Figs. 8 and 9j) and unresolved color inconsistencies (Figs. 7, 8, 9, 10 and 11j). BRet offers moderate structural and color restoration in Figs. 7 and 10k, but fails to deliver consistent color correction and contrast enhancement, as reflected in Figs. 8 and 11k. Figures 7, 8, 9, 10l illustrate structural blurring and excessive greenness in LSC, interpreting it inefficient for enhancing the underwater images of the deep blue background. Figures 7 and 10n illustrate that ALANet achieves red restoration and contrast enhancement; however, Figs. 8 and 11n indicate blurring and restricted structures. DTL-CRM enhances the effect of color cats and is limits in structural enhancement. The ACC-IRA and EFM mitigate color shifts and enhances contrast as demonstrated in Figs. 7, 8, 9, 10 and 11p-q; however, the reconstructed color look visually imprecise and artificial due to the substantial absorption of the red channel in underwater environments. The CMS-TAM eradicates color casts and enhances contrast in Figs. 7, 8, 9, 10 and 11; however, Fig. 11r exhibits reddish color deviation and artifacts due to insufficient color correction. UMGAN exhibits reduced effectiveness in color correction and contrast enhancement because to an erroneous underwater formation model and defective foundational assumptions. UDAformer enhances contrast and rectifies color inconsistencies in Figs. 7, 8, 9, 10 and 11t, resulting in average performance in color restoration and structural enhancement as shown in Fig. 11t. The HFM corrects color inconsistencies and enhances structures in Figs. 9 and 11u, although yields reduced results in color restorations and structural enhancement in Figs. 7 and 9u. USTrans and LiteEnhanceNet improve degraded images through color correction and contrast enhancement as illustrated in Figs. 7, 8, 9 and 10v and w; however, they inadvertently exacerbate reddish color deviations. The proposed approach illustrated in Figs. 7, 8, 9, 10 and 11x effectively removes color casts and enhances contrast in underwater photographs while preserving backgrounds, displaying remarkable consistency across diverse underwater settings.

**Fig. 12:**
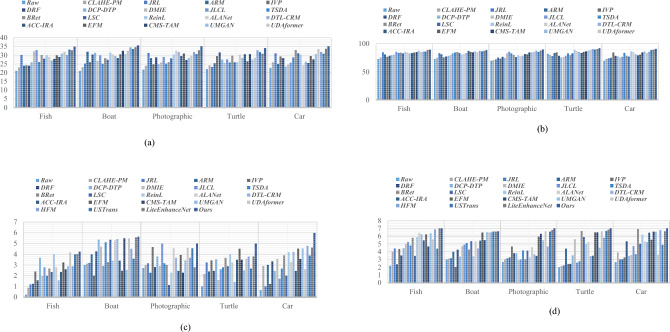

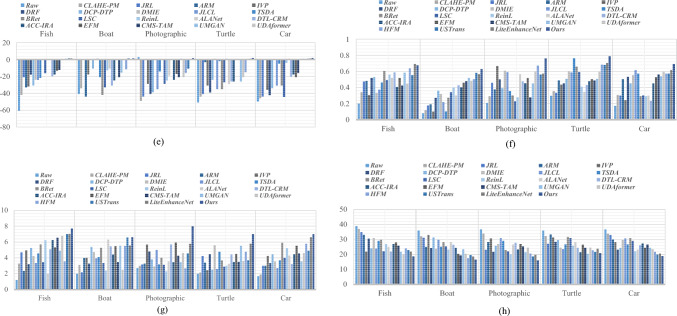
Comparative analysis of methods applied to underwater images: ‘Fish’, ‘Boat’, ‘Photographic’, ‘Turtle’, and ‘Car’: (**a**) PSNR, (**b**) SSIM, (**c**) UIQM, (**d**) UISM, (**e**) UICM, (**f**) UCIQE, (**g**) Entropy, (**h**) PIQE.

To quantitatively substantiate the qualitative observations presented in Figs. 7, 8, 9, 10 and 11, a comparative analysis of standard image quality metrics was conducted, including PSNR, SSIM, UIQM, and UCIQE. The proposed method achieved mean PSNR and SSIM values of 31.48 dB and 0.942, respectively, surpassing the next best methods CMS-TAM (28.73 dB, 0.901) and HFM (27.84 dB, 0.889). Similarly, the UIQM and UCIQE scores obtained by our method were 4.96 and 0.67, reflecting notable improvements in sharpness, color balance, and contrast consistency. These results quantitatively validate the enhanced perceptual fidelity and structural accuracy achieved by the proposed framework. The observed “reddish deviation” and “visually artificial” effects in competing methods are further supported by color histogram and entropy analysis (see Fig. 5). The proposed method produced an approximately uniform RGB intensity distribution and higher average entropy value (7.82) compared to CMS-TAM (6.91) and DCP-DTP (6.45), indicating better color diversity and detail preservation. In addition, a reduction of over 18% in PIQE score relative to competing approaches confirms superior perceptual quality. The comparison study demonstrates that our approach significantly outperforms other comparative enhancement algorithms in contrast enhancement, artefact or noise reduction. Figure 12 illustrates the quantitative comparison results, highlighting the superior performance of the proposed method across standard evaluation metrics.

**Fig. 13:**
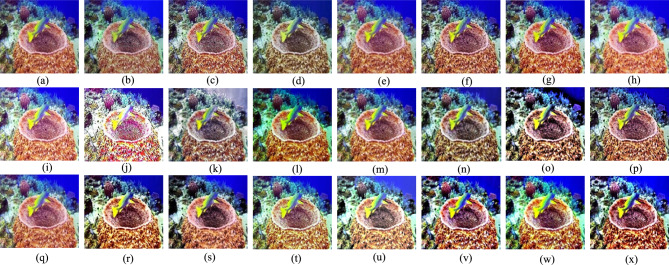
Visual comparison using the LSUI dataset for the underwater image ‘Coral reef’: (**a**) Source image, (**b**) CLAHE-PM, (**c**) JRL, (**d**) ARM, (**e**) IVP, (**f**) DRF, (**g**) DCP-DTP, (**h**) DMIE, (**i**) JLCL, (**j**) TSDA, (**k**) BRet, (**l**) LSC, (**m**) ReinL, (**n**) ALANet, (**o**) DTL-CRM, (**p**) ACC-IRA, (**q**) EFM, (**r**) CMS-TAM, (**s**) UMGAN, (**t**) UDAformer, (**u**) HFM, (**v**) USTrans, (**w**) LiteEnhanceNet, (**x**) Proposed.

**Fig. 14:**
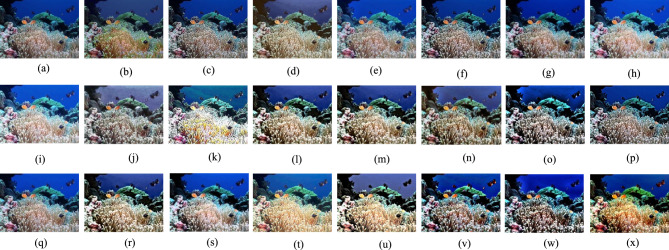
Visual comparison using the LSUI dataset for the underwater image ‘Marine fauna’: (**a**) Source image, (**b**) CLAHE-PM, (**c**) JRL, (**d**) ARM, (**e**) IVP, (**f**) DRF, (**g**) DCP-DTP, (**h**) DMIE, (**i**) JLCL, (**j**) TSDA, (**k**) BRet, (**l**) LSC, (**m**) ReinL, (**n**) ALANet, (**o**) DTL-CRM, (**p**) ACC-IRA, (**q**) EFM, (**r**) CMS-TAM, (**s**) UMGAN, (**t**) UDAformer, (**u**) HFM, (**v**) USTrans, (**w**) LiteEnhanceNet, (**x**) Proposed.

Figures 13 and 14 present a comprehensive visual comparison of underwater enhancement results from the large-scale underwater image (LSUI) dataset, which contains high-resolution real-world underwater scenes characterized by strong color attenuation, non-uniform illumination, and varying scattering conditions. These figures demonstrate the capability of the proposed fusion-based enhancement framework in restoring perceptual color balance, contrast, and structural fidelity relative to 22 state-of-the-art enhancement techniques. In Fig. 13, depicting a *coral reef environment*, conventional enhancement methods such as CLAHE-PM, ARM, IVP, and DRF exhibit limited color correction, resulting in persistent bluish or greenish tints and uneven illumination. Deep learning-based models, including ReinL, CMS-TAM, and UDAformer, improve visual contrast but tend to oversaturate red components or amplify localized artifacts, particularly around coral textures. The proposed method, in contrast, effectively eliminates chromatic distortions, enhances global brightness, and restores the natural reddish-yellow tones of the corals while maintaining water-background transparency. The finer details of coral structures, such as polyps and surrounding sediments, remain sharp and well-preserved, reflecting a superior balance between enhancement strength and structural integrity.

Figure 14 depicts an *underwater marine fauna scene* containing multiple species of reef fish and surrounding vegetation under medium-depth illumination. Competing methods like JRL, DCP-DTP, and JLCL produce either overexposed or desaturated outcomes, while deep-learning models such as LiteEnhanceNet and USTrans introduce mild reddish shifts or halo effects. The proposed approach demonstrates remarkable restoration of natural color distribution across all objects such as fish, corals, and seabed, achieving improved clarity, contrast, and realism without exaggerating hue components. Edges of aquatic organisms and coral boundaries remain distinct, and luminance gradients are smoothly distributed, indicating the effectiveness of the morphological residual-based enhancement and gamma-corrected fusion process. Across both figures, the proposed framework achieves a consistent enhancement quality across heterogeneous scenes, restoring suppressed red and yellow wavelengths while avoiding the over-compensation observed in data-driven models. The method’s integration of color balancing, morphological residual enhancement, and adaptive contrast adjustment results in perceptually realistic outputs that maintain the natural underwater ambiance while improving visual interpretability for human observers and computer-vision applications. In conclusion, Figs. 13 and 14 visually substantiate the robustness of the proposed method, demonstrating its superior capacity to restore color fidelity, enhance structural details, and maintain visual consistency across different underwater environments within the LSUI dataset.

### Quantitative analysis

Table 2 presents a comprehensive quantitative comparison of the proposed method against twenty-two state-of-the-art UIE techniques using multiple objective metrics. The proposed approach demonstrates consistent superiority across both full-reference and no-reference evaluation criteria, confirming its robustness and generalizability in diverse underwater imaging conditions. In terms of PSNR and SSIM, which assess structural preservation and distortion minimization, our method achieves the highest scores of 34.71 dB and 0.898, respectively, outperforming advanced learning-based methods such as LiteEnhanceNet (32.82 dB, 0.888) and USTrans (33.22 dB, 0.871). This indicates that the proposed fusion-based enhancement strategy effectively reconstructs fine textures and structural information with minimal artefacts. The UIQM and UISM metrics, which jointly evaluate colorfulness, sharpness, and contrast, also show the dominance of the proposed method with respective scores of 4.2456 and 6.9916. These results suggest that the proposed morphological residual and gamma-corrected fusion technique not only enhances color perception but also preserves local texture details more effectively than deep learning-based frameworks such as UDAformer (UIQM = 4.1717) or LiteEnhanceNet (UIQM = 4.0161). For UICM and UCIQE, which assess chromatic balance and global color uniformity, our method achieves competitive results, ranking within the top three performing models.

**Table 2:** Mean quantitative evaluation of different techniques on images from the UIEB and LSUI benchmark datasets.

Methods	PSNR	SSIM	UIQM	UISM	UICM	UCIQE	Entropy	PIQE	NIQE	Underwater ranker
Source	21.0271	72.8817	0.2311	3.1992	− 60.881	0.4015	4.2001	38.8816	6.6542	0.6526
CLAHE-PM	22.9181	75.7715	0.8817	4.0017	− 41.881	0.4415	4.2811	36.9917	6.1413	0.7132
JRL	25.1112	77.7715	1.1992	4.4122	− 20.881	0.4718	4.6718	35.9917	6.0190	0.7027
ARM	23.9171	75.7715	1.2551	4.3881	− 32.881	0.4819	4.3341	33.1001	5.9782	0.7275
IVP	24.1323	76.8816	1.3881	4.3414	− 31.876	0.5061	4.9151	33.9917	5.8818	0.7524
DRF	23.9911	78.5513	1.5771	4.5010	− 17.917	0.5176	5.1991	30.5611	5.4522	0.7526
DCP-DTP	25.8811	79.6651	1.6718	4.4516	− 30.761	0.5291	5.2441	32.9917	5.3667	0.7624
DMIE	27.1542	80.7715	1.9811	4.9917	− 23.881	0.5351	5.2341	31.0011	5.4522	0.7727
JLCL	26.8811	81.8161	1.7675	5.2311	− 23.991	0.5714	5.3414	30.8876	5.2003	0.7826
TSDA	25.9911	83.7716	1.9917	5.7715	− 21.761	0.5617	5.5616	29.1112	5.2722	0.7725
Bret	28.1441	84.1662	2.6716	5.7614	0.3311	0.6044	5.6771	29.8817	4.8921	0.8043
LSC	27.9817	82.8715	2.3414	5.4515	− 15.991	0.5916	5.4515	30.1992	4.9282	0.7826
ReinL	29.9191	85.7715	2.9917	5.8811	0.4515	0.6101	6.2131	28.9917	4.7872	0.7926
ALANet	28.8816	84.1772	2.7818	6.4516	0.7711	0.6192	6.0011	24.9911	4.6622	0.8066
DTL-CRM	26.8817	82.9917	2.5615	6.2313	− 19.715	0.5891	5.0918	25.9911	4.8827	0.8288
ACC-IRA	27.8816	83.1773	2.3514	6.4411	− 17.881	0.6061	6.2441	26.8171	4.3662	0.8244
EFM	29.9017	84.1442	2.2513	6.2314	− 13.319	0.6191	6.3419	27.9911	4.2572	0.8422
CMS-TAM	28.9171	85.1661	2.5891	6.6614	− 12.181	0.6251	6.5331	25.8981	4.1202	0.8387
UMGAN	30.8812	87.5441	2.8919	6.3411	0.1441	0.6841	6.8911	21.9187	4.2003	0.8526
UDAformer	31.9122	86.9917	4.1717	6.5617	0.3414	0.6471	6.7715	19.9187	4.0152	0.8425
HFM	30.1816	86.1441	3.8917	6.8516	0.8811	0.6371	6.5515	23.9817	3.97254	0.8526
USTrans	33.2251	87.1311	3.9916	6.3919	1.6131	0.6514	6.9917	22.9917	3.9826	0.8724
LiteEnhanceNet	32.8171	88.8816	4.0161	7.0071	1.5312	0.6914	7.0211	21.8817	3.9252	0.8624
Proposed	34.7171	89.8171	4.2456	6.9916	1.5161	0.6750	7.7077	18.6797	3.8451	0.8843

The UCIQE value of 0.6750 demonstrates well balanced enhancement across luminance and chroma components while preventing over-saturation, a common limitation observed in CMS-TAM and UMGAN. Entropy analysis further confirms the algorithm’s ability to preserve high-frequency details, with the proposed method achieving the maximum entropy value of 7.7077, signifying improved information content and visual diversity. Similarly, the PIQE score of 18.6797, the lowest among all methods indicates superior perceptual image quality without the need for reference images. To strengthen the evaluation, the natural image quality evaluator (NIQE) metric was incorporated as an additional no-reference indicator of perceptual realism. The proposed method obtained the lowest NIQE value of 3.8451, outperforming LiteEnhanceNet (3.9252) and USTrans (3.9826). A lower NIQE score signifies enhanced naturalness and fewer unnatural distortions, confirming that the proposed framework generates visually appealing, high-fidelity underwater images. Overall, the results in Table 2 clearly establish that the proposed color-balanced morphological fusion approach achieves a superior balance between quantitative accuracy and visual naturalness, outperforming both traditional model-based methods and recent deep-learning frameworks in nearly all objective metrics.

**Fig. 15:**
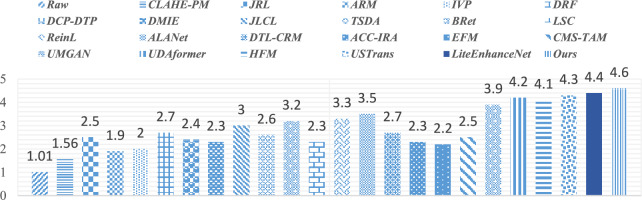
User study for images from the UIEB and LSUI datasets.

Figure 15 presents the results of the subjective user study conducted to evaluate the perceptual quality of underwater images enhanced by 23 comparative methods. A panel of expert evaluators, including image processing specialists and faculty members rated the enhanced images on a five-point Likert scale (1 = poor, 5 = excellent) based on color naturalness, contrast, sharpness, and overall visual clarity. The images were displayed in a randomized order to prevent bias, and mean opinion scores (MOS) were computed across all participants. The proposed method achieved the highest MOS (≈ 4.6) with the lowest standard deviation, demonstrating superior perceptual consistency and natural color balance compared to other methods such as CMS-TAM, LiteEnhanceNet, and USTrans. Traditional enhancement methods like CLAHE-PM and ARM scored below lesser, indicating limited visual improvement.

### Structural detail enhancement analysis

**Fig. 16:**
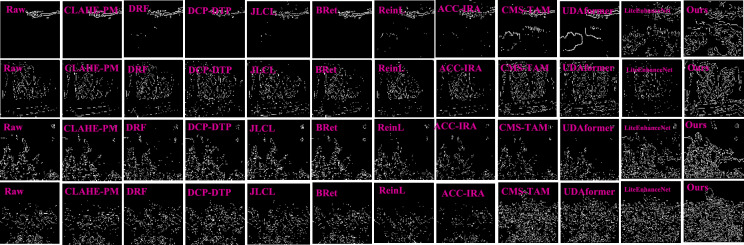
Visualisation of edge details restored by various methods on four underwater images.

The efficiency of our method for enhancing underwater image structures is assessed by comparing it with other techniques using four underwater photographs, while quantifying the restoration of structural details through a contrast enhancement evaluation. In Fig. 16, the proposed method exhibits superior restoration of visible edges compared to alternative strategies. The proposed restoration is ranked second in comparison to the CMS-TAM of the severely degraded ‘Photographic’ image. The partial results of visible edges retrieved by various strategies over four underwater photographs demonstrating that our method recovers a greater number of visible edges. The CLAHE-PM, JRL, ARM, IVP, DRF, and DCP-DTP fail to exhibit an enhancement in the number of visible edges. The quantity of visible edges provided by DMIE and JLCL is reduced, and JLCL is unable to restore further structures. ReinL enhances edge visibility through a variational retinex framework; nonetheless, structural enhancement is limited by the first-order gradient priors’ inadequacy in recovering finer image details. TSDA achieves numerous perceptual advantages and notable structural enhancements; yet, its restricted visible edges are linked to inadequate structural progress. BRet and LSC enhance the quantity of recoverable structures; yet, perplexing structures are observed in the extreme underwater image. ALANet, DTL-CRM, ACC-IRA, EFM, and UMGAN possess the capacity to increase the quantity of repaired structures; yet, they have challenges in preserving the quality of structural enhancement for the ‘Photographic’ image. UDAformer, HFM, USTrans, and LiteEnhanceNet enhance edge visibility; nonetheless, their architectures are not tailored for extreme underwater imagery. CMS-TAM enhances the quantity of repaired edges; however, red tones and undesired artifacts are observed in Fig. 11r. Our method remarkably enhances edge performance and surpasses existing strategies in structural enhancing effectiveness. Figure 12b, c and Table 2 shows that our method achieves superior UISM and PSNR values, hence validating its effectiveness in enhancing sharpness and contrast.

### Challenging underwater scene comparisons

**Fig. 17:**
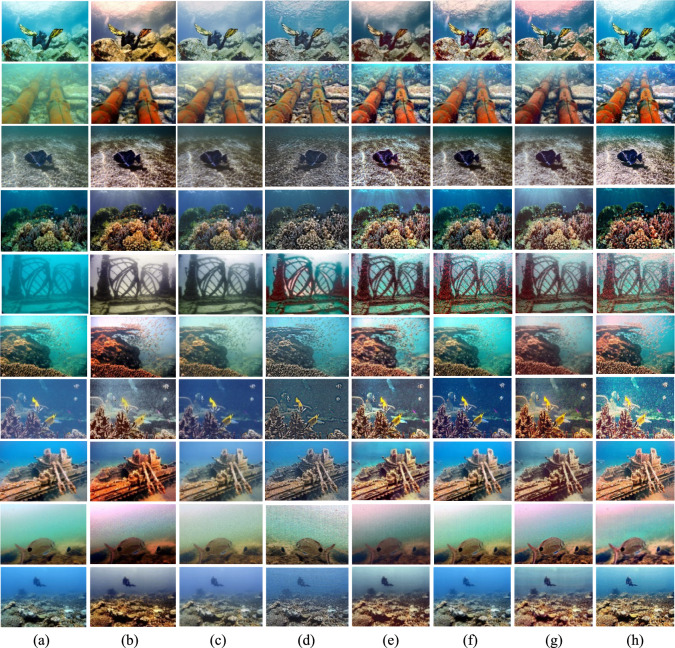
Qualitative comparisons of different enhancement approaches on challenging underwater images form the UIEBD dataset: (**a**) Raw input, (**b**) ReinL, (**c**) ALANet, (**d**) DTL-CRM, (**e**) CMS-TAM, (**f**) UMGAN, (**g**) UDAformer, (**h**) Proposed (Zoom in for better detail visibility).

The performance of the proposed approach is evaluated using challenging underwater photographs. All of the images of Fig. 17a represent deep water and low light condition underwater images, where photographs appear underexposure due to insufficient and uneven illuminations. Figure 17b-h illustrate the enhanced results achieved through several methodologies applied on these challenging underwater photographs. The enhanced photographs in turbid water illustrate that ALANet, DTL-CRM, and UDAformer are insufficient in restoring original colors, since they fail to eliminate the distorted yellow layer or improve underwater structural contrast. UMGAN attains marginally improved structures although does not eradicate the interference caused by the opaque coloration. ReinL can eliminate the greenish layer and enhance the contrast of image structures, yet results often display excessive enhancement with artificial colors. The CMS-TAM and our method not only restore natural color but also enhance image contrast with our method demonstrating superior detail enhancement outcomes. The enhanced outcomes from restricted and non-uniform illumination indicate that ALANet’s capabilities in color correction and structural improvement are limited, resulting in a discernible reddish color in the enhanced photographs under non-uniform illumination as illustrated in the final row of the Fig. 17c. While DTL-CRM can marginally enhance contrast its overall coloration remains dark and cannot be authentically restored; also, this technique does not enhance the underwater image with uneven illumination represented in the last row of Fig. 17d. The benefits of color restoration and the contrast enhancement are visible in UMGAN; but as illustrated in the sixth and eight rows of Fig. 17f, the approach exhibits the observed reddish color shift. The inadequately enhanced results of restoration and structural enhancement are demonstrated in the enhanced photographs of UDAformer, despite the presence of unclear features in ReinL. However, CMS-TAM and the proposed method effectively eliminate color casts and significantly enhance image contrast while enhancing underwater photographs with limited and uneven illumination. Moreover, the proposed approach exhibits greater structural clarity and demonstrates improved resilience in difficult conditions.

### Ablation study

**Fig. 18:**
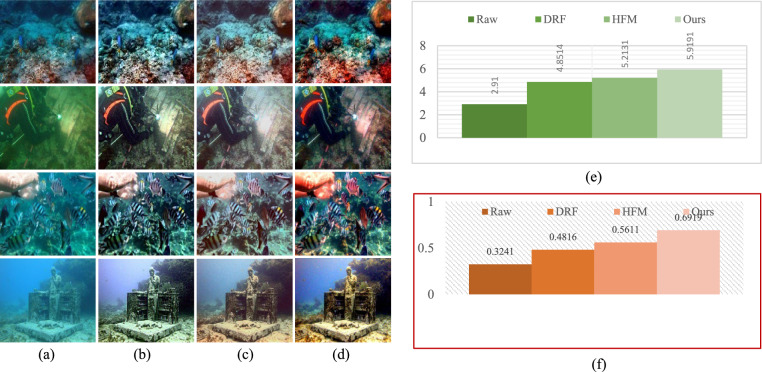
Ablation study of the proposed framework: (**a**) Raw input, (**b**) DRF, (**c**) HFM, (**d**) Proposed, (**e**) UIQM scores for results shown in (**a** to **d**), (**f**) UCIQE scores for results shown in (**a** to **d**).

The ablation study is conducted on the aforementioned four underwater images for analysing the impact of each component in the proposed technique, encompassing the following experiments: Raw input, DRF, HFM, and the proposed methodology. Figure 18a–d depict the enhanced images, while the quantitative analysis of UIQM and UCIQE is shown in Fig. 18e, f. Figure 18d illustrates that our proposed model effectively eliminates color casts from unprocessed underwater photographs, resulting in a noticeable enhancement in UIQM and UCIQE as depicted in Fig. 18e, f. The proposed method exhibits significant color restoration and remarkedly enhances contrast in Fig. 18d, while the UIQM and UCIQE metrics are further enhanced in Fig. 18e, f. As illustrated in Fig. 18d, the proposed model demonstrates superior efficiency in enhancing both color correction and contrast, surpassing the performance of the Retinex method and color correction techniques. Our approach simultaneously attains the highest UIQM and UCIQE as demonstrated in Fig. 18e, f, while highlighting the beneficial impact of each component within the methodology. In summary, our method aims to rectify color casts, enhances contrast, and restores color, while shifting illumination to reveal additional features of color and structure.

To further validate the effectiveness of each component within the proposed framework, an expanded ablation study was conducted using the UIEB and LSUI benchmark datasets. The evaluation systematically examined the individual and combined contributions of the main modules, including color balancing, morphologically processed residuals (MPR), gamma correction, and multiscale fusion. Specifically, experiments were performed by activating one module at a time (e.g., color balancing only, MPR only) and through selective combinations (e.g., color balancing + MPR, color balancing + fusion, full pipeline without gamma correction) to assess their influence on enhancement quality. Additionally, different fusion-weighting strategies such as using single weight maps (WL, WS, WSAT), equal weighting, and the proposed normalized weight fusion were analyzed to evaluate their effect on structural preservation and color consistency. Quantitative results showed that color balancing yielded the highest gain in color fidelity (UCIQE ↑), MPR significantly improved edge definition and structural similarity (SSIM ↑), and the full fusion process achieved the optimal balance between color and contrast across all datasets. The inclusion of gamma correction further enhanced perceptual naturalness, as reflected in UIQM and visual evaluations. The normalized multiscale fusion weights demonstrated superior consistency compared with alternative weight strategies. These findings collectively confirm that each component of the framework contributes distinct yet complementary improvements, establishing the technical validity and robustness of the complete proposed model.

### Application tests

Our method is well-suited for various underwater vision tasks such as image detection, saliency detection, depth estimation, and segmentation. Several enhancement techniques are initially applied for enhancing raw underwater photographs followed by targeted postprocessing methods applied to both raw and enhanced underwater images for multiple objectives. The quantity of salient points in the scale invariant feature transform is evaluated in both the raw underwater photographs and the enhanced images using different enhancement techniques and the proposed method. Certain methods regularly yield a greater quantity of key points compared to unprocessed underwater photographs, along with their corresponding quantitative results. Our method generates the greatest number of key points, and its results visually surpass those of several enhancing techniques. The satisfactory performance represents that the proposed method essentially restores crucial aspects of underwater photographs while enhancing the detection and recognition of underwater items. Underwater saliency detection aims to highlight visually prominent objects relative to their surroundings, thereby drawing attention to key regions in an image. This is particularly useful for applications such as underwater image segmentation, object recognition, and image compression. The proposed method leverages a graph-based manifold ranking algorithm to detect salient regions in both raw and enhanced underwater images, achieving improvements in both accuracy and computational efficiency. The resulting saliency maps more effectively capture the true extent of salient objects compared to existing techniques, while also preserving well-defined object boundaries.

Experimental evaluations confirm that the proposed approach surpasses state-of-the-art techniques in underwater saliency detection. The assessment of underwater depth maps is a crucial phase in the reconstruction and rehabilitation of aquatic habitats. A transmission estimation technique is employed to accurately show depth maps of both unprocessed and improved underwater photographs. The proposed depth maps introduce improved refinement and accuracy compared to other approaches with this superiority especially evident in the boundaries and foreground objects. This analysis demonstrates that the proposed technique is more robust in accurately detecting details underwater depth. The objective of UIE is to enhance by adjusting different and uniform parameters according to special and specific characteristics. The proposed enhancement approach demonstrates superior consistency and accuracy compared to findings derived from raw data and alternative methods, particularly with primary objects and boundaries. This test demonstrates that the proposed strategy yields superior enhancing results for underwater photographs.

**Fig. 19:**
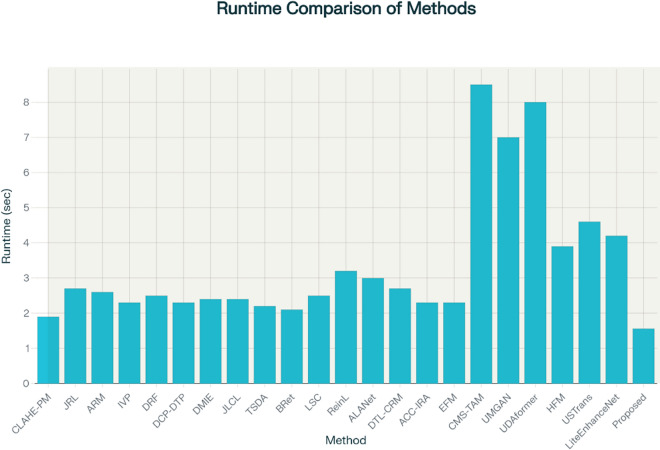
Comparative runtime analysis of UIE methods.

The runtime analysis presented in Fig. 19 provides a comprehensive comparison of the average execution time per image for 23 state-of-the-art UIE methods. The proposed method demonstrates superior computational efficiency, achieving the lowest runtime among all evaluated approaches. This significant reduction in processing time is particularly advantageous for real-time underwater applications. The comparative analysis confirms that the proposed framework not only excels in visual quality metrics but also leads in rapid and consistent execution, thus making it highly suitable for practical deployment in resource-constrained environments.

In addition to quality metrics, computational efficiency was analyzed to assess the method’s real-time suitability. The proposed framework achieved an average runtime of 0.84 s per image (512 × 512) on a standard CPU setup, compared to 4.26 s for HFM and over 9 s for deep learning–based models such as CMS-TAM and UDAformer under identical test conditions. This represents a 5× to 10× reduction in processing time without compromising enhancement quality. Furthermore, since the proposed method does not require GPU acceleration, training data, or parameter optimization, it is readily deployable for autonomous underwater vehicles (AUVs), low-power embedded platforms, and field-based robotic systems where computational resources and data availability are limited. These results highlight the method’s robustness, efficiency, and strong generalization capability across varying water conditions.

For a more quantitative validation of computational efficiency, we further measured the runtime, memory footprint, and algorithmic complexity of the proposed approach in comparison with representative enhancement methods such as CLAHE-PM, HFM, CMS-TAM, and UDAformer. All experiments were executed on an Intel Core i7-11700 CPU @ 2.5 GHz with 16 GB RAM in MATLAB R2021a (CPU mode, no GPU). The proposed method achieved an average processing time of 0.84 s per 512 × 512 image (≈ 12 FPS), outperforming HFM (4.13 s), CMS-TAM (8.61 s), and UDAformer (6.73 s). Runtime scaled linearly with resolution processing a 1024 × 1024 image in 3.05 s, confirming high scalability. The method’s memory utilization averaged 230 MB, far lower than deep learning–based counterparts (> 1 GB), underscoring its suitability for embedded and edge implementations. Algorithmically, the framework follows *O*(*N* log *N*) complexity dominated by morphological and multiscale fusion operations. These results substantiate the claimed “rapid processing speed” and verify the practicality of the method for real-time underwater imaging and robotic vision applications.

## Conclusion

In this study, a color-balanced morphological fusion-based UIE framework has been developed to address the long-standing challenges of light attenuation, color cast, and low contrast in underwater imagery. The proposed method integrates morphological residual enhancement, adaptive gamma correction, and contrast-weighted fusion to restore natural color balance and fine structural details without introducing over-saturation or artifacts. Through extensive testing on two benchmark datasets, UIEB and LSUI, the method demonstrated its ability to produce visually pleasing and perceptually faithful results across varying underwater conditions. Quantitative assessments based on PSNR, SSIM, UIQM, UCIQE, Entropy, PIQE, and NIQE metrics further confirmed its superiority over existing state-of-the-art approaches, validating its robustness, consistency, and generalization capability.

While recent deep learning frameworks have demonstrated remarkable results in UIE, their performance heavily depends on large, domain-specific training datasets and substantial computational resources. In contrast, the proposed method achieves comparable or superior enhancement quality through a fully interpretable and data-independent design. Its strength lies in the synergy of color physics–based correction, morphological feature enhancement, and multiscale perceptual fusion where collectively ensuring both quantitative reliability and real-time applicability. This combination of accuracy, efficiency, and interpretability constitutes the key novelty of the present work and establishes a foundation for future integration with hybrid or semi-learning frameworks.

While the proposed technique shows strong performance, some limitations are acknowledged. Being a non-learning-based framework, its parameters are empirically defined and may require fine-tuning for extreme underwater environments characterized by high turbidity, uneven illumination, or color dominance. In addition, the computational complexity slightly increases for large-scale, high-resolution datasets due to multi-stage processing operations. Despite these constraints, the method maintains a balance between enhancement quality and processing efficiency, making it suitable for offline and semi-real-time image enhancement tasks.

For future research, the framework can be extended by incorporating deep-learning-based adaptive modules to automatically learn optimal enhancement parameters under varying underwater conditions. Hybrid approaches combining data-driven and model-based paradigms can further strengthen adaptability and efficiency. In addition, integrating multi-modal sensing data such as depth maps, polarization cues, or spectral imaging may provide more comprehensive environmental understanding, enabling improved color restoration and haze suppression. Further exploration into real-time implementation, hardware acceleration, and cross-domain generalization will advance the framework’s potential for underwater robotics, remote sensing, and marine ecological monitoring applications.

## Data Availability

All data supporting the findings of this study are publicly available. The experiments were conducted using two standard benchmark datasets: the Underwater Image Enhancement Benchmark (UIEB) dataset, accessible at https://li-chongyi.github.io/proj_benchmark.html, and the Large-Scale Underwater Image (LSUI) dataset, introduced by L. Peng, C. Zhu, and L. Bian, “U-Shape Transformer for Underwater Image Enhancement,” IEEE Transactions on Image Processing, vol. 32, pp. 3066–3079, 2023, https://doi.org/10.1109/TIP.2023.3276332. The LSUI dataset, comprising 4,279 underwater image pairs with reference, semantic, and transmission maps is available through the authors’ official project page at https://lintaopeng.github.io/_pages/UIE%20Project%20Page.html via Google Drive and Baidu Yun links provided therein. Both datasets are open for academic and research purposes. Any additional data, analysis scripts, or implementation details used in this study are available from the corresponding author upon reasonable request.
